# Risk factors for suicide in adults: systematic review and meta-analysis of psychological autopsy studies

**DOI:** 10.1136/ebmental-2022-300549

**Published:** 2022-09-26

**Authors:** Louis Favril, Rongqin Yu, Abdo Uyar, Michael Sharpe, Seena Fazel

**Affiliations:** 1 Faculty of Law and Criminology, Ghent University, Ghent, Belgium; 2 Department of Psychiatry, University of Oxford, Oxford, UK

**Keywords:** Suicide & self-harm, Adult psychiatry

## Abstract

**Question:**

Effective prevention of suicide requires a comprehensive understanding of risk factors.

**Study selection and analysis:**

Five databases were systematically searched to identify psychological autopsy studies (published up to February 2022) that reported on risk factors for suicide mortality among adults in the general population. Effect sizes were pooled as odds ratios (ORs) using random-effects models for each risk factor examined in at least three independent samples.

**Findings:**

A total of 37 case–control studies from 23 countries were included, providing data on 40 risk factors in 5633 cases and 7101 controls. The magnitude of effect sizes varied substantially both between and within risk factor domains. Clinical factors had the strongest associations with suicide, including any mental disorder (OR=13.1, 95% CI 9.9 to 17.4) and a history of self-harm (OR=10.1, 95% CI 6.6 to 15.6). By comparison, effect sizes were smaller for other domains relating to sociodemographic status, family history, and adverse life events (OR range 2–5).

**Conclusions:**

A wide range of predisposing and precipitating factors are associated with suicide among adults in the general population, but with clear differences in their relative strength.

**PROSPERO registration number:**

CRD42021232878.

**WHAT IS ALREADY KNOWN ON THIS TOPIC**

- Psychological autopsy provides one validated approach to determine factors that contribute to suicide. Previous reviews of this literature have focused on a selected number of risk factors.

**WHAT THIS STUDY ADDS**

- We have synthesised a wide range of risk factors for suicide, which allows for a comparison of their relative risks. Sources of heterogeneity were examined by meta-regression and subgroup analyses.

**HOW THIS STUDY MIGHT AFFECT RESEARCH, PRACTICE OR POLICY**

- Suicide prevention should combine public health approaches with interventions that target high-risk groups, including those with mental disorders, a history of self-harm, and recent adverse life events.

## Background

Suicide is a global public health concern, accounting for over 700 000 deaths annually.^1^ Identifying factors associated with suicide can improve risk stratification and help target interventions for high-risk groups.^2^ One widely used approach to investigating suicide risk factors at the individual level is the psychological autopsy method,^3^ which involves collecting information about the person who has died by suicide through standardised interviews with proxy informants (such as family members) and, when available, examination of medical and coronial records. This retrospective approach aims to construct a comprehensive picture of the clinical and psychosocial circumstances that contributed to the suicide. In doing so, psychological autopsies allow for examination of a wider range of risk factors, and in more detail, than possible in register-based studies which rely on data that is routinely collected for administrative purposes.^4^ To our knowledge, five previous reviews have quantitatively summarised findings from psychological autopsy studies,^5–9^ but these have been limited in scope (primarily focusing on mental disorders) and samples (including selected populations such as psychiatric patients). The most recent meta-analysis, based on studies published up to 2016, only examined associations with mood and substance use disorders.^9^ Synthesising the full range of risk factors reported in the psychological autopsy literature by adopting a uniform analytical approach would allow for a direct comparison of their associations with suicide.

## Objectives

We have conducted a systematic review and meta-analysis to provide an up-to-date and comprehensive synthesis of psychological autopsy studies comparing adults in the general population who died by suicide with those who did not. We aimed to improve the precision of effect sizes of previously identified risk factors, to delineate associations not examined in prior meta-analyses and to compare estimates across risk factor domains. Findings could assist clinicians to prioritise interventions based on modifiable risk factors and their relative strengths, researchers to consider evidence gaps and policy makers to target resources most effectively.

## Study selection and analysis

This systematic review and meta-analysis was conducted following the Preferred Reporting Items for Systematic Reviews and Meta-analyses guidelines^10^ (online supplemental table S1). The study protocol was pre-registered with PROSPERO.

10.1136/ebmental-2022-300549.supp1 Supplementary data

### Search strategy

We systematically searched five electronic databases (PubMed, Global Health, Embase, Web of Science and PsycINFO) for psychological autopsy studies published from inception to 28 February 2022. Keywords were identical for all databases: (suicid*) AND (autops*). No language restrictions were set. Bibliographical searches were complemented by manual searches of reference lists of included studies and previous systematic reviews.^5–9^

### Selection criteria

One researcher (AU) screened titles and abstracts of the retrieved articles for eligibility. Full-text publications were then screened with an additional reviewer (LF) and disagreements were resolved by consensus. Primary studies using the psychological autopsy method to examine risk factors for death by suicide in adults from the general population were eligible for inclusion. The following inclusion criteria were set: the psychological autopsy study (1) has a quantitative observational design, (2) includes predominantly adults aged 18–65 years, (3) is based on a general population sample, (4) includes suicide mortality as outcome and (5) provides data for a control group of general population individuals either living in the community (living control group) or who died from causes other than suicide (deceased control group). The inclusion of studies with controls who died by causes other than suicide is consistent with previous reviews of psychological autopsy studies.^5–9^ We excluded studies (1) with qualitative and case designs, (2) that examined self-harm or attempted suicide as outcome, (3) without proxy interviews available for all suicide cases, (4) with selected samples (eg, soldiers) or specific age groups (eg, exclusively adolescents or older adults) and (5) including a high-risk control group (eg, psychiatric patients). Studies excluded based on these sample criteria are listed in online supplemental table S2.

### Data extraction

Data extraction was done independently by two authors (LF and AU) using a predetermined form listing relevant study characteristics (ie, publication year, country and sample size), sample details (ie, age, sex and type of control group) and risk factor estimates. Extraction sheets were cross-checked for consistency and discrepancies were resolved by discussion within the research team. When study characteristics were unclear, the authors of the respective primary papers were contacted. When multiple publications on the same or overlapping study population were available, information on risk factors was extracted from the investigation with the largest sample. Data were only extracted from overlapping publications when a new risk factor was reported. For example, a 2002 Chinese investigation analysed risk factors for suicide in 519 cases and 536 controls,^11^ for which an update was published in 2010 that covered an overlapping sample of 895 cases and 701 controls.^12^ In this case, we extracted data from the 2010 article (with the largest sample) and only included data from the 2002 investigation if an additional risk factor was reported. For brevity, one main reference is provided for each unique study (see online supplemental table S3 for a list of all included publications).

### Data analysis

Crude ORs and their 95% CIs were extracted as reported or calculated from available prevalence data in the paper (eg, 2×2 contingency tables). The majority (78%) of studies also included adjusted estimates derived from multivariable models. As adjustments were inconsistent across studies, ranging from basic demographic characteristics to psychiatric diagnoses, we selected crude estimates for the main analysis in order to reduce heterogeneity due to non-comparability. In sensitivity analyses, however, we compared adjusted to crude effect sizes when reported in the same study.

Risk factors were categorised into four broad domains: sociodemographic, clinical, family history and adverse life events. Similar risk factors within a particular domain were grouped. For example, all alcohol-related exposures (eg, alcohol dependence, abuse and misuse) were coded as alcohol use disorder.^6^ A recent meta-analysis indicated that combining these variables into a single measure does not lead to a significant loss of information.^9^ All mental disorders were based on standardised diagnostic criteria. Additionally, adverse life events were grouped into three temporal categories: within the past month, 3 months and 6 months. In a secondary analysis, we estimated the crude prevalence of the strongest risk factors within an individual domain.

Risk of bias was assessed using the Newcastle-Ottawa Scale for case–control studies.^13^ Studies were rated on three criteria (ie, selection, comparability and exposure) with a total of eight items (eg, representativeness of the cases, comparability of cases and controls and ascertainment of exposure), resulting in an overall score between 0 and 9. A maximum score of 9 points indicates low risk of bias.

Meta-analyses were conducted using the *metan* command in Stata IC (V.13). To ensure accuracy and reliability of obtained estimates, meta-analysis was performed only for risk factors that were examined in at least three independent samples.^14^ For all analyses, a random-effects model (using the inverse-variance method) was generated to account for the anticipated heterogeneity between studies. Heterogeneity was estimated using the I² statistic, which specifies the percentage of variation in effect sizes underlying the different studies. Considerable heterogeneity across studies is indicated by an I² value of 75% and over.^15^ The degree to which methodological differences between studies moderated the associations between risk factors and suicide was investigated using univariate meta-regression models (using the *metareg* command). Meta-regression was considered for risk factors when there were 10 or more samples in the meta-analysis.^15^ Categorical variables examined in meta-regression were type of control group (living vs deceased), data source for control subjects (proxy informants vs controls themselves) and region (low/middle-income vs high-income). Risk of bias score (0–9), proportion of men in the sample (%) and sample size (n) were included as continuous covariates. We were not able to include mean age at death as a continuous covariate in meta-regression since a third (32%) of studies did not provide such information. The presence of publication bias was examined by visual inspection of funnel plots^16^ and by applying Egger’s test^17^ for variables examined in at least 10 independent samples.^15^

Post hoc, we conducted subgroup analyses by type of control group (living vs deceased) and sensitivity analyses excluding studies in which controls acted as their own informants.

## Findings

### Study characteristics

Our systematic search of the literature yielded 4284 unique records for screening, from which 231 full-text reports were examined for eligibility (online supplemental figure S1). A total of 37 studies reported in 97 publications met our inclusion criteria (table 1). This discrepancy between the number of studies and publications is partly explained by one Chinese investigation that resulted in more than 20 publications on the same sample (online supplemental table S3). Studies were conducted across 23 countries (21 [57%] in high-income countries) and published between 1994 and 2021. All included studies had a case–control design, with sample sizes ranging from 45 to 1596 (median=216, IQR=194–400). Collectively, studies included a total of 12 734 individuals, of which 7101 (56%) were controls and 5633 (44%) were suicide cases, with an average of 152 suicides per study. The mean age of the suicide group was 39.8 years (SD=10.9) and 71% were men. All but three (92%) studies included both men and women. Twenty-nine (78%; n=9194) studies recruited living community controls and eight (22%; n=3540) used a control group of people who died from causes other than suicide (mostly accidents and sudden deaths). In five (14%; n=2419) studies, information on the living control group was collected directly from (a portion of) the control subjects themselves, rather than by interviewing proxy informants.

**Table 1:** Study characteristics

Study	Country	Case group			Control group*		NOS
		n	Age, M (range)	Men (%)	n	Type	
Almasi *et al* (2009)^e1^	Hungary	194	43 (30–62)	80.9	194	Living†	5
Altindag *et al* (2005)^e2^	Turkey	26	24 (12–62)	15.4	25	Living	9
Anton-San-Martin *et al* (2013)^e3^	Spain	40	56 (19–90)	67.5	80	Living	7
Appleby *et al* (1999)^e4^	England	84	27 (13–34)	81.0	64	Living	6
Arafat *et al* (2021)^e5^‡	Bangladesh	100	26 (9–75)	49.0	100	Living	7
Beautrais (2001)^e6^‡	New Zealand	202	37 (14–88)	77.7	984	Living	7
Chachamovich *et al* (2015)^e7^	Canada	120	23	82.5	120	Living	4
Chen *et al* (2006)^e8^‡	China	150	39 (15–59)	64.0	150	Living	7
Cheng *et al* (2000)^e9^‡	Taiwan	113	NR	61.5	226	Living	8
de la Vega Sanchez *et al* (2020)^e10^	Spain	192	54	100	81	Deceased	4
De Leo *et al* (2013)^e11^‡	Australia	261	≥35	75.1	182	Deceased	6
Foster *et al* (1999)^e12^	Ireland	117	16–76	79.5	117	Living	7
Gururaj *et al* (2004)^e13^	India	269	75% 16–49	64.3	269	Living†	7
Jia *et al* (2014)^e14^‡	China	200	61 (11–93)	57.5	200	Living	7
Jollant *et al* (2014)^e15^	Philippines	15	15–64	73.3	30	Living	5
Khan *et al* (2008)^e16^	Pakistan	100	NR	83.0	100	Living	7
Kim *et al* (2003)^e17^‡	Canada	115	29 (18–65)	100	82	Living	8
Kodaka *et al* (2017)^e18^‡	Japan	102	≥20	69.6	334	Living	7
Kolves *et al* (2006)^e19^‡	Estonia	427	48	80.3	427	Living†	7
Kurihara *et al* (2009)^e20^	Indonesia	60	41 (13–87)	63.3	120	Living	9
Manoranjitham *et al* (2010)^e21^	India	100	42	59.0	100	Living	6
Martiello *et al* (2019)^e22^	Italy	91	≥25	80.2	270	Living	7
Morales & Martinez (2010)^e23^	Colombia	101	28	69.3	112	Deceased	7
Nicolas *et al* (2016)^e24^‡	Canada	42	37	50.0	42	Living	5
Overholser *et al* (2012)^e25^	United States	148	47	78.4	257	Deceased	4
Owens *et al* (2003)^e26^	England	100	≥18	67.0	100	Living	6
Page *et al* (2014)^e27^	Australia	84	18–34	84.5	250	Living	7
Palacio *et al* (2007)^e28^‡	Colombia	108	29 (19–43)	80.6	108	Deceased	6
Politakis *et al* (2017)^e29^	Slovenia	90	49	70.0	90	Living	4
Rasouli *et al* (2019)^e30^	Iran	40	39 (19–75)	80.0	40	Living†	7
Ross *et al* (2017)^e31^	Australia	126	25–44	100	68	Deceased	6
Schneider *et al* (2006)^e32^‡	Germany	163	50	64.4	396	Living†	6
Tong & Phillips (2010)^e33^‡	China	895	42 (12–94)	50.7	701	Deceased	5
Vijayakumar & Rajkumar (1999)^e34^	India	100	≥15	55.0	100	Living	7
Zhang *et al* (2004)^e35^‡	China	66	46	72.7	66	Living	5
Zhang *et al* 2010^e36^‡	China	392	27 (15–34)	54.6	416	Living	7
Zonda (2006)^e37^	Hungary	100	52	67.0	100	Deceased	7

Note. References are provided in online supplemental table S3.

*Matched with the case group for age and sex in all but five studies.^e6,e10,e11,e25,e33^

†Control subjects acted as their own informants.

‡Multiple publications for this study.

NOS, Newcastle-Ottawa Scale score; NR, not reported.

### Quality assessment

Of a maximum of 9 points, the mean quality score of case–control studies was 6.4 (SD=1.3, range 4–9). Twenty-one (57%) studies had a score of 7 points or more, indicating low risk of bias (online supplemental table S4). Across studies, non-response was a key weakness. Many investigations with considerable differences in response rates (>10%) between cases and controls did not clarify reasons for this discrepancy and therefore scored conservatively for this domain.

### Risk estimates

We identified 40 risk factors that were examined in at least three independent samples (table 2). For significant associations, pooled ORs ranged from 2.2 to 4.0 in the sociodemographic domain. The strongest risk factors identified were social isolation (OR=4.0, 95% CI 2.1 to 7.7), unemployment (OR=3.8, 95% CI 2.7 to 5.2) and low socioeconomic status (OR=2.8, 95% CI 1.8 to 4.2). Risk factors within the family history domain were a family history of mental disorder (OR=5.2, 95% CI 1.9 to 14.1), suicide (OR=3.7, 95% CI 2.3 to 5.7) and attempted suicide (OR=2.8, 95% CI 1.5 to 5.0).

**Table 2:** Pooled estimates of risk factors for suicide, by domain

	Samples (k)	Cases (n)	Controls (n)	OR (95% CI)	z	I²
*Sociodemographic domain*						
Social isolation	10	1549	1613	4.0 (2.1 to 7.7)	4.2	95
Unemployment	25	4010	4439	3.8 (2.7 to 5.2)	8.1	79
Low socioeconomic status	4	284	450	2.8 (1.8 to 4.2)	4.9	0
Low education	19	3099	4474	2.7 (2.1 to 3.5)	8.2	64
Single/not married	21	2773	3576	2.4 (1.8 to 3.2)	6.4	74
Low income	6	1530	2317	2.4 (1.6 to 3.6)	4.2	74
Living alone	15	2748	3014	2.3 (1.5 to 3.4)	4.0	77
Not religious	12	1383	1550	2.2 (1.4 to 3.5)	3.4	77
Not having children	6	1026	979	1.3 (0.9 to 1.8)	1.4	61
*Family history domain*						
Mental disorder	6	415	800	5.2 (1.9 to 14.1)	3.2	82
Suicide	14	2018	2268	3.7 (2.3 to 5.7)	5.7	73
Suicide attempt	7	1638	1789	2.8 (1.5 to 5.0)	3.4	82
*Clinical domain*						
Any mental disorder	28	4085	4368	13.1 (9.9 to 17.4)	17.8	76
Depression	22	3432	3805	11.0 (7.3 to 16.5)	11.5	77
Schizophrenia spectrum disorder	17	3056	4165	7.8 (4.5 to 13.5)	7.4	63
Bipolar disorder	8	1220	1485	4.6 (2.1 to 10.1)	3.8	33
Substance use disorder	21	3525	4621	3.7 (2.8 to 5.0)	8.7	71
Alcohol use disorder	18	2777	4277	3.2 (2.3 to 4.4)	6.5	71
Drug use disorder	6	1163	2098	3.0 (1.7 to 5.4)	3.7	20
Anxiety disorder	17	2997	4199	2.5 (1.7 to 3.5)	4.8	52
Dysthymia	6	964	1566	2.4 (1.4 to 4.1)	3.2	36
Any personality disorder (PD)	13	1437	1634	6.8 (4.7 to 9.8)	10.3	42
Borderline PD	7	754	1009	9.0 (5.6 to 14.4)	9.0	0
Paranoid PD	5	519	807	6.2 (3.5 to 11.2)	6.1	0
Dependent PD	4	393	739	6.1 (2.5 to 15.1)	3.9	0
Avoidant PD	5	526	701	3.9 (1.4 to 11.1)	2.5	69
Antisocial PD	5	515	715	3.4 (2.0 to 6.1)	4.3	0
Psychiatric treatment	11	2171	2972	10.5 (7.4 to 14.9)	13.1	41
History of self-harm	26	3466	4582	10.1 (6.6 to 15.6)	10.5	77
Previous suicide attempt	22	2971	4107	8.5 (5.3 to 13.4)	9.1	77
Smoking	4	412	660	4.3 (2.3 to 7.9)	4.6	67
Physical illness	16	2963	3253	2.9 (2.4 to 3.6)	11.2	19
*Adverse life events*						
Relationship conflict	10	1338	2318	5.0 (3.3 to 7.6)	7.8	73
Legal problems	6	889	1769	4.8 (2.4 to 9.4)	4.5	75
Family-related conflict	9	1154	1364	4.5 (2.0 to 10.3)	3.6	92
Abuse/victimisation	6	823	803	3.5 (2.4 to 5.0)	6.8	0
Financial problems	15	1805	2261	2.8 (2.0 to 4.0)	5.9	65
Early separation from parents	4	344	676	2.7 (1.6 to 4.5)	3.7	5
Work/school-related conflict	11	1614	2565	1.8 (1.1 to 2.8)	2.5	84
Bereavement	7	889	1122	1.2 (0.5 to 3.1)	0.4	86
Timing of life events						
Within past month	7	1140	1136	10.4 (7.1 to 15.3)	12.1	19
Within past 6 months	3	402	402	5.3 (1.8 to 15.9)	3.0	91
Within past 3 months	4	613	613	2.9 (1.6 to 5.0)	3.7	72

**Figure 1:**
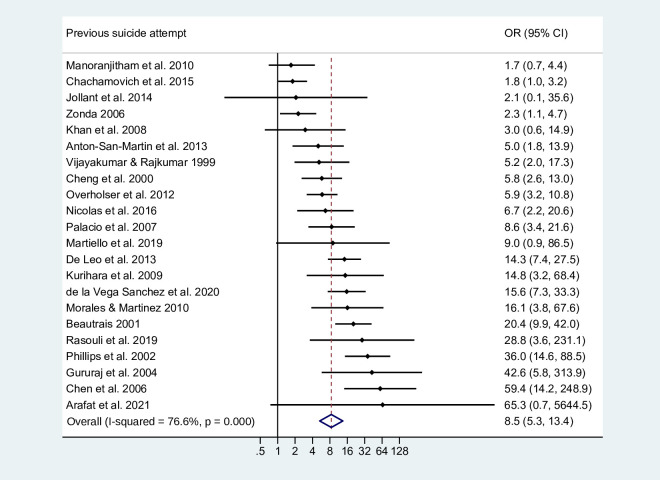
Previous suicide attempt as a risk factor for suicide. The dots and lines represent the ORs and corresponding 95% CIs from each primary study. The diamond denotes the pooled summary effect size and CI from random-effects meta-analysis. References are provided in online supplemental table S3.

Within the clinical domain, a history of self-harm (OR=10.1, 95% CI 6.6 to 15.6) and a previous suicide attempt (OR=8.5, 95% CI 5.3 to 13.4; figure 1) were both strongly associated with suicide. We found strong associations for any mental disorder (OR=13.1, 95% CI 9.9 to 17.4) and any personality disorder (OR=6.8, 95% CI 4.7 to 9.8). By diagnosis, depression had the strongest association with suicide (OR=11.0, 95% CI 7.3 to 16.5), followed by borderline personality disorder (OR=9.0, 95% CI 5.6 to 14.4) and schizophrenia spectrum disorder (OR=7.8, 95% CI 4.5 to 13.5). Risk of suicide was comparable for alcohol use disorder (OR=3.2, 95% CI 2.3 to 4.4) and drug use disorder (OR=3.0, 95% CI 1.7 to 5.4).

For adverse life events, relationship conflict (OR=5.0, 95% CI 3.3 to 7.6), legal problems (OR=4.8, 95% CI 2.4 to 9.4) and family-related conflict (OR=4.5, 95% CI 2.0 to 10.3) had the strongest associations with suicide. By timing, adverse life events occurring within the previous month increased the risk of suicide 10-fold (OR=10.4, 95% CI 7.1 to 15.3).

The prevalence of the strongest risk factor within each domain is listed in online supplemental table S5—all of which were more than 40% in people who died by suicide. Seven in 10 (71.1%) cases had a mental disorder at the time of death compared with 22.2% of controls. In addition, a previous suicide attempt was documented for 28.5% of cases and 6.0% of controls.

### Sensitivity analysis

Comparisons between crude and adjusted estimates are provided in online supplemental table S6 for 21 risk factors. Adjusted ORs were smaller than crude ORs for all but three factors examined, with an average reduction of 19% in risk estimates (ranging from +48% to −61%). The largest reductions were found in the clinical domain. For example, the crude OR for a history of self-harm (OR=18.1, 95% CI 13.2 to 24.6) was two times larger than its adjusted counterpart (aOR=9.1, 95% CI 6.0 to 13.7). Similar differences were found for any personality disorder (OR=10.7 vs aOR=4.2) and any mental disorder (OR=12.1 vs aOR=7.7). Differences between crude and adjusted estimates should, however, be interpreted with caution because many studies exclusively reported significant associations from the multivariable models, which likely results in biased comparisons between estimates.

### Meta-regression

We examined six variables as possible sources of between-study heterogeneity in estimates for 21 risk factors where there were at least 10 samples (online supplemental table S7). Type of control group (living vs deceased) was significantly associated with heterogeneity for 7 (33%) out of all 21 risk factors studied: marital status, living alone, any mental disorder, schizophrenia spectrum disorder, substance use disorder, alcohol use disorder, and any personality disorder. We found that studies including a deceased control group reported smaller effect sizes for these risk factors compared with those including living controls. Furthermore, source of data for the control group (proxy informants vs control individuals themselves) explained some of the between-study heterogeneity only for schizophrenia spectrum disorder and a history of self-harm. Results indicate larger effect sizes for these two risk factors in studies where control subjects were interviewed directly (instead of proxy respondents). Income region was a moderator only for the association between depression and suicide, with a smaller effect observed in samples from high-income countries. No consistent associations for between-study heterogeneity were found for sample size, risk of bias and proportion of men.

### Publication bias

We found evidence of publication bias for eight risk factors (online supplemental table S7). Egger’s test was significant for social isolation (p=0.001), unemployment (p=0.015), living alone (p=0.044), any mental disorder (p=0.009), depression (p=0.071), substance use disorder (p=0.006), any personality disorder (p=0.006) and financial problems (p=0.061). Funnel plots for these variables are shown in online supplemental figures S2–S9.

## Conclusions and implications

In this systematic review and meta-analysis of 37 psychological autopsy studies comparing 5633 adults who died by suicide with 7101 controls, we have synthesised findings on a wide range of risk factors for suicide in the general population. Our work adds to the evidence base in three ways.

First, previous reviews of psychological autopsy studies^5–9^ have not captured the breadth of individual-level risk factors for suicide, and instead mainly focused on the contribution of mental disorders to suicide risk. In contrast, we meta-analysed effect sizes for a broader range of risk factors, which allows for comparison between risk factor domains. Specifically, across the 40 risk factors under study, the strongest associations with suicide were found in the clinical domain. The presence of any mental disorder was associated with more than a 10-fold increase in the odds of suicide, as was a history of self-harm. By comparison, effect sizes (ranging from 2 to 5) were smaller for other domains relating to sociodemographic factors, family history, and adverse life events. Absolute rates were also high, with 71% of suicide decedents reported to have a mental disorder at the time of death and 29% having previously attempted suicide.

Second, we found considerable variation in risk estimates within each domain. For example, although it is well known that having a mental disorder is a strong risk factor for suicide,^2^ there were clear differences between diagnoses in their relative strength. Effect sizes ranged from 2 for dysthymia and 3 for substance use disorders to 11 for depression. To our knowledge, we have for the first time quantified associations for individual personality disorders with suicide, with risk estimates in the range of 3 (antisocial personality disorder) to 9 (borderline personality disorder). In keeping, we also found such variation in effect sizes for adverse life events. Legal problems (such as contact with the criminal justice system) and interpersonal conflict had the strongest associations with suicide risk. The negative effect that exposure to stressful life events has on an individual’s overall functioning and life expectations may, at least in part, explain these findings.^18^ As expected, we found timing to be of significance, with adverse events occurring in the month before death being the most important. Other studies have found a dose–response relationship between the number of events experienced and the risk of suicide.^18^

Third, we have investigated possible explanations for the observed heterogeneity in risk estimates. Specifically, for a third of risk factors examined in meta-regression, significantly smaller effect sizes were observed in studies that included a control group of individuals who died from causes other than suicide, relative to studies with living controls. This finding was corroborated in post hoc subgroup analyses (online supplemental table S8). Comparing suicide cases with controls who have died by other external causes such as accidents may generate smaller or no differences between groups because of overlapping risk factors^19^ and because prematurely deceased controls may be at increased risk of suicide themselves.^4^ In addition, some deaths classified as accidents will include probable suicides,^20^ further reducing differences between cases and controls. This observation that heterogeneity is associated with study design underscores the need for a standardised approach to conducting psychological autopsies. We recommend that the optimal control group would be living individuals. Researchers need to consider, though, the implications of using living controls on the timing of risk factors, which may underestimate the effects of certain factors. To ensure comparability between groups, it has been recommended that information on living controls should equally be obtained by means of proxy-based interviews.^21^ In meta-regression analyses, however, we found that how data was collected for the control group (ie, whether proxy informants or controls themselves were interviewed) did not moderate the strength of associations for most risk factors. Post hoc sensitivity analyses confirmed this finding, showing no major change in the magnitude of estimates after removal of studies in which controls acted as their own informants (online supplemental table S9).

### Strengths and limitations

Strengths of this review include the use of a quantitative synthesis and examination of a wide range of risk factors not limited to mental disorders. Several limitations should also be noted. First, the strength of the reported associations is likely to be overestimated as we did not account for confounding, and the risk factors examined are not independent from one another (eg, psychiatric comorbidity). In sensitivity analyses, we found that the majority of adjusted estimates were materially smaller than crude ones, with an average reduction of a fifth in effect sizes and up to half for clinical risk factors. However, because different studies used contrasting approaches to adjustment, which would make adjusted estimates difficult to compare, our main analysis focused on crude effect sizes. Future work should consider confounding more carefully, examine a fuller range of sociodemographic confounds when assessing clinical factors, and use consistent and replicable approaches. Second, we identified considerable heterogeneity for about one-third of all risk estimates. Although a high degree of heterogeneity can be expected in meta-analyses of observational studies,^22^ pooled effect sizes should be interpreted with caution, and ranges should be considered. This heterogeneity is likely caused by varying instruments used to assess risk factors. Third, we retained all eligible studies in our analyses irrespective of their methodological quality, which may have influenced the results—though there was little evidence to suggest that study quality was associated with heterogeneity in meta-regression. Fourth, we were unable to examine risk factors for men and women separately because most primary studies did not stratify analyses by sex; future work should investigate this. Fifth, our findings should not be generalised to adolescents and older adults as we only focused on working-age adults. Risk factors are known to vary by age groups^2^ and might have a different impact according to when they occur in someone’s life (eg, unemployment and physical illness).

In addition, there are several limitations inherent to the psychological autopsy approach,^23–25^ including issues relating to the choice (and selective non-response) of control subjects and the reliance on proxy reports—the latter being prone to recall bias and measurement error. While register-based studies may minimise such biases, they are more restricted in scope since many potential risk factors are not captured in routinely collected administrative records (eg, recent life events, social isolation, religious beliefs and self-harm not resulting in service contact). Although the method of psychological autopsy has been criticised for overestimating the contribution of mental disorders to suicide,^25^ risk estimates for diagnostic categories in the current review (in particular when limited to studies using living controls) were largely similar in magnitude to those reported in a recent meta-analysis of record linkage studies.^22^ In contrast, prospective cohort studies of suicide mortality in the general population—which allow for a more reliable measurement of exposures—tend to report more conservative risks for sociodemographic factors, adverse life events, family history of mental disorder, and depression.^26–28^ Among other reasons, effects might be smaller in prospective studies owing to long follow-up periods, whereas psychological autopsies focus on the time window close to death.^9^ Notwithstanding its limitations, the psychological autopsy method can complement other study designs to identify suicide risk factors, allow for triangulation of evidence, and may be the approach of choice in settings where population-based registers are not available or when prospective studies are not feasible.

### Clinical implications

The main implication of this review is that suicide is associated with many factors across clinical, life events, family history, and sociodemographic domains—and within a particular domain, there are differential associations with suicide. Consistent with a stress-diathesis model,^29^ this underscores the need to understand suicide as the result of a cumulation of multiple risk factors,^2^ some of which are predisposing (such as sociodemographic background and family history) and some precipitating (such as incident diagnoses and life events). Consequently, suicide prevention should combine strategies aimed at a general reduction in population risk (eg, through means restriction) with interventions that focus on high-risk groups, such as people with severe mental illness or recently hospitalised for self-harm.^30–32^

Our findings underline the importance of both assessment and treatment of mental disorders in healthcare settings.^33^ Screening and assessment in itself will not improve outcomes, unless it leads to effective intervention.^34^ This approach differs from that in general population settings, where many people at elevated risk of suicide do not access healthcare services and links to effective interventions may not be possible or timely. Rather, high-risk groups should be a focus for prevention as they are likely to already be accessing healthcare services, such as individuals who have attempted suicide, especially if they have underlying mental disorders.^35^ Interventions to consider include psychosocial treatment following self-harm, particularly cognitive behavioural approaches, which can reduce risk of repetition.^36^ Brief interventions such as safety planning^37^ have also been found to prevent future suicidal behaviour. Another high-risk group with a treatment gap is people with depression, for whom effective pharmacological and psychosocial treatments are available^38^ and assessment of suicide risk can be improved by considering somatic^39^ and psychiatric^40^ comorbidities. Further, our findings highlight the need for assessment of suicide risk that incorporates multiple factors, and their interactions, in prediction models.^41^ Previous research found single factors, including self-harm, to be poor predictors of subsequent suicide.^2^

### Conclusion

We found that a wide range of predisposing and precipitating risk factors are associated with suicide among adults in the general population, but to differing degrees. Our findings highlight a number of modifiable risk factors and suggest that suicide prevention should combine strategies aimed at a general reduction in population risk with interventions that focus on high-risk groups. As part of this, healthcare services at national, regional and local levels can review the extent and quality of clinical and psychosocial interventions to reduce suicide risk.

## Data Availability

Data are available upon reasonable request.
