# Supplementary material for: Risk factors for suicide in adults: systematic review and meta-analysis of psychological autopsy studies
Source: Evid Based Ment Health. 2022 Sep 26;25(4):148–55. doi: 10.1136/ebmental-2022-300549 (PMC9685708; doi:10.1136/ebmental-2022-300549)

Table S1. PRISMA checklist.

Section and topic	Item	Checklist item	Location where item is reported
**TITLE**			
Title	1	Identify the report as a systematic review.	Page 1
**ABSTRACT**			
Abstract	2	See the PRISMA 2020 for Abstracts checklist.	Page 2
**INTRODUCTION**			
Rationale	3	Describe the rationale for the review in the context of existing knowledge.	Page 3
Objectives	4	Provide an explicit statement of the objective(s) or question(s) the review addresses.	Page 3
**METHODS**			
Eligibility criteria	5	Specify the inclusion and exclusion criteria for the review and how studies were grouped for the syntheses.	Page 4
Information sources	6	Specify all databases, registers, websites, organisations, reference lists and other sources searched or consulted to identify studies. Specify the date when each source was last searched or consulted.	Page 4
Search strategy	7	Present the full search strategies for all databases, registers and websites, including any filters and limits used.	Page 4
Selection process	8	Specify the methods used to decide whether a study met the inclusion criteria of the review, including how many reviewers screened each record and each report retrieved, whether they worked independently, and if applicable, details of automation tools used in the process.	Page 4
Data collection process	9	Specify the methods used to collect data from reports, including how many reviewers collected data from each report, whether they worked independently, any processes for obtaining or confirming data from study investigators, and if applicable, details of automation tools used in the process.	Page 4-5
Data items	10a	List and define all outcomes for which data were sought. Specify whether all results that were compatible with each outcome domain in each study were sought (e.g. for all measures, time points, analyses), and if not, the methods used to decide which results to collect.	Page 4
	10b	List and define all other variables for which data were sought (e.g. participant and intervention characteristics, funding sources). Describe any assumptions made about any missing or unclear information.	Page 4-5
Study risk of bias assessment	11	Specify the methods used to assess risk of bias in the included studies, including details of the tool(s) used, how many reviewers assessed each study and whether they worked independently, and if applicable, details of automation tools used in the process.	Page 5
Effect measures	12	Specify for each outcome the effect measure(s) (e.g. risk ratio, mean difference) used in the synthesis or presentation of results.	Page 5
Synthesis methods	13a	Describe the processes used to decide which studies were eligible for each synthesis (e.g. tabulating the study intervention characteristics and comparing against the planned groups for each synthesis (item #5)).	Page 4
	13b	Describe any methods required to prepare the data for presentation or synthesis, such as handling of missing summary statistics, or data conversions.	Page 5

Favril L, *et al. Evid Based Ment Health* 2022; 25:148–155. doi: 10.1136/ebmental-2022-300549

Section and topic	Item	Checklist item	Location where item is reported
	13c	Describe any methods used to tabulate or visually display results of individual studies and syntheses.	Page 5
	13d	Describe any methods used to synthesize results and provide a rationale for the choice(s). If meta-analysis was performed, describe the model(s), method(s) to identify the presence and extent of statistical heterogeneity, and software package(s) used.	Page 5
	13e	Describe any methods used to explore possible causes of heterogeneity among study results (e.g. subgroup analysis, meta-regression).	Page 5-6
	13f	Describe any sensitivity analyses conducted to assess robustness of the synthesized results.	Page 5-6
Reporting bias assessment	14	Describe any methods used to assess risk of bias due to missing results in a synthesis (arising from reporting biases).	Page 5-6
Certainty assessment	15	Describe any methods used to assess certainty (or confidence) in the body of evidence for an outcome.	Page 6
**RESULTS**			
Study selection	16a	Describe the results of the search and selection process, from the number of records identified in the search to the number of studies included in the review, ideally using a flow diagram.	Page 6, Figure S1
	16b	Cite studies that might appear to meet the inclusion criteria, but which were excluded, and explain why they were excluded.	Table S2
Study characteristics	17	Cite each included study and present its characteristics.	Table 1<br>Table S3
Risk of bias in studies	18	Present assessments of risk of bias for each included study.	Page 6-7<br>Table S4
Results of individual studies	19	For all outcomes, present, for each study: (a) summary statistics for each group (where appropriate) and (b) an effect estimate and its precision (e.g. confidence/credible interval), ideally using structured tables or plots.	Figure 2
Results of syntheses	20a	For each synthesis, briefly summarise the characteristics and risk of bias among contributing studies.	Page 6-8
	20b	Present results of all statistical syntheses conducted. If meta-analysis was done, present for each the summary estimate and its precision (e.g. confidence/credible interval) and measures of statistical heterogeneity. If comparing groups, describe the direction of the effect.	Page 7<br>Table 2
	20c	Present results of all investigations of possible causes of heterogeneity among study results.	Page 7-8<br>Table A7
	20d	Present results of all sensitivity analyses conducted to assess the robustness of the synthesized results.	Page 8-10<br>Table S8<br>Table S9
Reporting biases	21	Present assessments of risk of bias due to missing results (arising from reporting biases) for each synthesis assessed.	Page 8
Certainty of evidence	22	Present assessments of certainty (or confidence) in the body of evidence for each outcome assessed.	NA

Favril L, *et al. Evid Based Ment Health* 2022; 25:148–155. doi: 10.1136/ebmental-2022-300549

Section and topic	Item	Checklist item	Location where item is reported
**DISCUSSION**			
Discussion	23a	Provide a general interpretation of the results in the context of other evidence.	Page 8-10
	23b	Discuss any limitations of the evidence included in the review.	Page 10-11
	23c	Discuss any limitations of the review processes used.	Page 10-11
	23d	Discuss implications of the results for practice, policy, and future research.	Page 11-12
**OTHER INFORMATION**			
Registration and protocol	24a	Provide registration information for the review, including register name and registration number, or state that the review was not registered.	Page 3
	24b	Indicate where the review protocol can be accessed, or state that a protocol was not prepared.	Page 3
	24c	Describe and explain any amendments to information provided at registration or in the protocol.	NA
Support	25	Describe sources of financial or non-financial support for the review, and the role of the funders or sponsors in the review.	Page 1
Competing interests	26	Declare any competing interests of review authors.	Page 1
Availability of data, code and other materials	27	Report which of the following are publicly available and where they can be found: template data collection forms; data extracted from included studies; data used for all analyses; analytic code; any other materials used in the review.	Page 1

Table S2. Selection of studies that were excluded on the basis of sample characteristics.

Selected population: occupational group
Bhise, M.C., & Behere, P.B. (2016). Risk factors for farmers' suicides in central rural India: matched case-control psychological autopsy study. *Indian Journal of Psychological Medicine, 38*(6), 560–566.
Farberow, N.L., Kang, H.K., & Bullman, T.A. (1990). Combat experience and postservice psychosocial status as predictors of suicide in Vietnam veterans. *Journal of Nervous and Mental Disease, 178*(1), 32–37.
Hawton, K., Simkin, S., Rue, J., Haw, C., Barbour, F., Clements, A., et al. (2002). Suicide in female nurses in England and Wales. *Psychological Medicine, 32*(2), 239–250.
Nock, M.K., Dempsey, C. L., Aliaga, P.A., Brent, D.A., Heeringa, S.G., Kessler, R.C., et al. (2017). Psychological autopsy study comparing suicide decedents, suicide ideators, and propensity score matched controls: results from the study to assess risk and resilience in service members (Army STARRS). *Psychological Medicine, 47*(15), 2663–2674.
Thoresen, S., & Mehlum, L. (2006). Suicide in peacekeepers: risk factors for suicide versus accidental death. *Suicide and Life-Threatening Behavior, 36*(4), 432–442.
**Specific age group: children and adolescents**
Brent, D.A., Baugher, M., Bridge, J., Chen, T., & Chiappetta, L. (1999). Age- and sex-related risk factors for adolescent suicide. *Journal of the American Academy of Child and Adolescent Psychiatry, 38*(12), 1497–1505.
Freuchen, A., Kjelsberg, E., Lundervold, A.J., & Groholt, B. (2012). Differences between children and adolescents who commit suicide and their peers: a psychological autopsy of suicide victims compared to accident victims and a community sample. *Child and Adolescent Psychiatry and Mental Health, 6*: 1.
Gould, M.S., Fisher, P., Parides, M., Flory, M., & Shaffer, D. (1996). Psychosocial risk factors of child and adolescent completed suicide. *Archives of General Psychiatry, 53*(12), 1155–1162.
Renaud, J., Berlim, M.T., McGirr, A., Tousignant, M., & Turecki, G. (2008). Current psychiatric morbidity, aggression/impulsivity, and personality dimensions in child and adolescent suicide: a case-control study. *Journal of Affective Disorders, 105*(1-3), 221–228.
Shafii, M., Carrigan, S., Whittinghill, J. R., & Derrick, A. (1985). Psychological autopsy of completed suicide in children and adolescents. *American Journal of Psychiatry, 142*(9), 1061–1064.
**Specific age group: older adults**
Chiu, H.F., Yip, P.S., Chi, I., Chan, S., Tsoh, J., Kwan, C.W., et al. (2004). Elderly suicide in Hong Kong—a case-controlled psychological autopsy study. *Acta Psychiatrica Scandinavica, 109*(4), 299–305.
Conwell, Y., Duberstein, P.R., Hirsch, J.K., Conner, K.R., Eberly, S., & Caine, E.D. (2010). Health status and suicide in the second half of life. *International Journal of Geriatric Psychiatry, 25*(4), 371–379.
Harwood, D., Hawton, K., Hope, T., & Jacoby, R. (2001). Psychiatric disorder and personality factors associated with suicide in older people: a descriptive and case-control study. *International Journal of Geriatric Psychiatry, 16*(2), 155–165.
Preville, M., Hebert, R., Boyer, R., Bravo, G., & Seguin, M. (2005). Physical health and mental disorder in elderly suicide: a case-control study. *Aging & Mental Health, 9*(6), 576–584.
Waern, M., Runeson, B.S., Allebeck, P., Beskow, J., Rubenowitz, E., Skoog, I., et al. (2002). Mental disorder in elderly suicides: a case-control study. *American Journal of Psychiatry, 159*(3), 450–455.
Zhou, L., Wang, G., Jia, C., & Ma, Z. (2019). Being left-behind, mental disorder, and elderly suicide in rural China: a case-control psychological autopsy study. *Psychological Medicine, 49*(3), 458–464.

High-risk control group (adults)

Cavanagh, J.T., Owens, D.G., & Johnstone, E.C. (1999). Suicide and undetermined death in south east Scotland: a case-control study using the psychological autopsy method. *Psychological Medicine, 29*(5), 1141–1149.

Giupponi, G., Innamorati, M., Baldessarini, R.J., De Leo, D., de Giovannelli, F., Pycha, R., et al. (2018). Factors associated with suicide: case-control study in South Tyrol. *Comprehensive Psychiatry, 80*, 150–154.

Gray, D., Coon, H., McGlade, E., Callor, W.B., Byrd, J., Viskochil, J., et al. (2014). Comparative analysis of suicide, accidental, and undetermined cause of death classification. *Suicide and Life-Threatening Behavior, 44*(3), 304–316.

Pompili, M., Innamorati, M., Di Vittorio, C., Baratta, S., Masotti, V., Badaracco, A., et al. (2014). Unemployment as a risk factor for completed suicide: a psychological autopsy study. *Archives of Suicide Research, 18*(2), 181–192.

High-risk control group (adolescents)

Gagnon, A., Davidson, S.I., Cheifetz, P.N., Martineau, M., & Beauchamp, G. (2009). Youth suicide: a psychological autopsy study of completers and controls. *Vulnerable Children and Youth Studies, 4*(1), 13–22.

Houston, K., Hawton, K., & Shepperd, R. (2001). Suicide in young people aged 15–24: a psychological autopsy study. *Journal of Affective Disorders, 63*(1-3), 159–170.

Portzky, G., Audenaert, K., & van Heeringen, K. (2009). Psychosocial and psychiatric factors associated with adolescent suicide: a case-control psychological autopsy study. *Journal of Adolescence, 32*(4), 849–862.

Table S3. References of psychological autopsy studies included in the meta-analysis.

Study	Publication(s)
e1	Almasi, K., Belso, N., Kapur, N., Webb, R., Cooper, J., Hadley, S., et al. (2009). Risk factors for suicide in Hungary: a case-control study. *BMC Psychiatry*, *9*: 45.
e2	Altindag, A., Ozkan, M., & Oto, R. (2005). Suicide in Batman, southeastern Turkey. *Suicide and Life-Threatening Behavior*, *35*(4), 478–482.
e3	Anton-San-Martin, J.M., Sanchez-Guerrero, E., Perez-Costilla, L., Labajos-Manzanares, M., Diego-Otero, Y., Comino-Ballesteros, R., et al. (2013). Risk and protective factors in suicide: a case control study using the psychological autopsy. *Anales De Psicologia, 29*(3), 810–815.
e4	Appleby, L., Cooper, J., Amos, T., & Faragher, B. (1999). Psychological autopsy study of suicides by people aged under 35. *British Journal of Psychiatry*, *175*(2), 168–174.
e5	Arafat, S.M.Y., Mohit, M.A., Mullick, M.S.I., Kabir, R., & Khan, M.M. (2021). Risk factors for suicide in Bangladesh: case-control psychological autopsy study. *BJPsych Open*, *7*(1): e18.
	Arafat, S.M.Y., Khan, M.A.S., Knipe, D., & Khan, M.M. (2021). Population attributable fractions of clinical and social risk factors for suicide in Bangladesh: finding from a case-control psychological autopsy study. *Brain and Behavior, 11*(12): e2409.
	Arafat, S.Y., Mohit, M.A., Mullick, M.I., Khan, M.S., Khan, M.M. (2021). Suicide with and without mental disorders: findings from psychological autopsy study in Bangladesh. *Asian Journal of Psychiatry, 61*: 102690.
e6	Beautrais, A.L. (2001). Suicides and serious suicide attempts: two populations or one? *Psychological Medicine, 31*(5), 837–845.
	Beautrais, A.L. (2003). Suicide and serious suicide attempts in youth: a multiple-group comparison study. *American Journal of Psychiatry, 160*(6), 1093–1099.
	Conner, K.R., Beautrais, A.L., & Conwell, Y. (2003). Moderators of the relationship between alcohol dependence and suicide and medically serious suicide attempts: analyses of Canterbury Suicide Project data. *Alcoholism: Clinical and Experimental Research*, *27*(7), 1156–1161.
e7	Chachamovich, E., Kirmayer, L.J., Haggarty, J.M., Cargo, M., Mccormick, R., & Turecki, G. (2015). Suicide among Inuit: results from a large, epidemiologically representative follow-back study in Nunavut. *Canadian Journal of Psychiatry, 60*(6), 268–275.
e8	Chen, E.Y.H., Chan, W.S.C., Wong, P.W.C., Chan, S.S.M., Chan, C.L.W., Law, Y.W., et al. (2006). Suicide in Hong Kong: a case-control psychological autopsy study. *Psychological Medicine, 36*(6), 815–825.
	Chan, S.S., Chiu, H.F., Chen, E.Y., Chan, W.S., Wong, P.W., Chan, C.L., et al. (2009). Population-attributable risk of suicide conferred by axis I psychiatric diagnoses in a Hong Kong Chinese population. *Psychiatric Services*, *60*(8), 1135–1138.
	Chan, W.S., Yip, P.S., Wong, P.W., & Chen, E.Y. (2007). Suicide and unemployment: what are the missing links? *Archives of Suicide Research, 11*(4), 327–335.
	Law, Y.W., Wong, P.W., & Yip, P.S. (2010). Suicide with psychiatric diagnosis and without utilization of psychiatric service. *BMC Public Health, 10*: 431.
	Law, Y.W., Wong, P.W.C., & Yip, P.S.F. (2015). Health and psychosocial service use among suicides without psychiatric illness. *Social Work, 60*(1), 65–74.

	Law, Y.W., Yip, P.S., Zhang, Y., & Caine, E.D. (2014). The chronic impact of work on suicides and under-utilization of psychiatric and psychosocial services. *Journal of Affective Disorders, 168*, 254–261
	Wong, P.W.C., Chan, W.S.C., Chen, E.Y.H., Chan, S.S.M., Law, Y.W., & Yip, P.S.F. (2008). Suicide among adults aged 30–49: a psychological autopsy study in Hong Kong. *BMC Public Health, 8*: 147.
e9	Cheng, A.T.A., Chen, T.H.H., Chen, C.C., & Jenkins, R. (2000). Psychosocial and psychiatric risk factors for suicide: case-control psychological autopsy study. *British Journal of Psychiatry*, *177*(4), 360–365.
	Cheng, A.T.A. (1995). Mental illness and suicide: a case-control study in East Taiwan. *Archives of General Psychiatry, 52*(7), 594–603.
	Cheng, A.T.A., Mann, A.H., & Chan, K.A. (1997). Personality disorder and suicide: a case-control study. *British Journal of Psychiatry, 170*(5), 441–446.
	Lee, C.S., Chang, J.C., & Cheng, A.T.A. (2002). Acculturation and suicide: a case-control psychological autopsy study. *Psychological Medicine, 32*(1), 133–141.
e10	de la Vega Sanchez, D., Guija, J.A., Perez-Moreno P., Kelly, S.A., Santos, M., Oquendo, M.A., et al. (2020). Association of religious activity with male suicide deaths. *Suicide & Life-Threatening Behavior, 50*(2), 449–460.
e11	De Leo, D., Draper, B. M., Snowdon, J., & Kolves, K. (2013). Suicides in older adults: a case-control psychological autopsy study in Australia. *Journal of Psychiatric Research, 47*(7), 980–988.
	De Leo, D., Draper, B.M., Snowdon, J., & Kolves, K. (2013). Contacts with health professionals before suicide: missed opportunities for prevention? *Comprehensive Psychiatry, 54*(7), 1117–1123.
	Draper, B., Kolves, K., De Leo, D., & Snowdon, J. (2014). A controlled study of suicide in middle-aged and older people: personality traits, age, and psychiatric disorders. *Suicide & Life-Threatening Behavior*, *44*(2), 130–138.
	Kolves, K., Draper, B.M., Snowdon, J., & De Leo, D. (2017). Alcohol-use disorders and suicide: results from a psychological autopsy study in Australia. *Alcohol, 64*, 29–35.
e12	Foster, T., Gillespie, K., McLelland, R., & Patterson, C. (1999). Risk factors for suicide independent of DSM-III-R axis I disorder: case-control psychological autopsy study in Northern Ireland. *British Journal of Psychiatry*, *175*(2), 175–179.
e13	Gururaj, G., Isaac, M.K., Subbakrishna, D.K., & Ranjani, R. (2004). Risk factors for completed suicides: a case-control study from Bangalore, India. *International Journal of Injury Control and Safety Promotion, 11*(3), 183–191.
e14	Jia, C.X., Wang, L.L., Xu, A.Q., Dai, A.Y., & Qin, P. (2014). Physical illness and suicide risk in rural residents of contemporary China: a psychological autopsy case-control study. *Crisis*, *35*(5), 330–337.
	Lu, C.F., Jia, C.X., Xu, A.Q., Dai, A.Y., & Qin, P. (2013). Psychometric characteristics of Chinese version of Barratt Impulsiveness Scale-11 in suicides and living controls of rural China. *Omega, 66*(3), 215–229.
e15	Jollant, F., Malafosse, A., Docto, R., & Macdonald, C. (2014). A pocket of very high suicide rates in a non-violent, egalitarian, and cooperative population of South-East Asia. *Psychological Medicine, 44*(11), 2323–2329.
e16	Khan, M.M., Mahmud, S., Karim, M.S., Zaman, M., & Prince, M. (2008). Case-control study of suicide in Karachi, Pakistan. *British Journal of Psychiatry, 193*(5), 402–405.

e17	Kim, C., Lesage, A., Seguin, M., Chawky, N., Vanier, C., Lipp, O., & Turecki, G. (2003). Patterns of co-morbidity in male suicide completers. *Psychological Medicine, 33*(7), 1299–1309.
	Lesage, A. D., Boyer, R., Grunberg, F., Vanier, C., Morissette, R., Menard-Buteau, C., & Loyer, M. (1994). Suicide and mental disorders: a case-control study of young men. *American Journal of Psychiatry, 151*(7), 1063–1068.
	Ernst, C., Lalovic, A., Lesage, A., Seguin, M., Tousignant, M., & Turecki, G. (2004). Suicide and no axis I psychopathology. *BMC Psychiatry, 4*: 7.
e18	Kodaka, M., Matsumoto, T., Takai, M., Yamauchi, T., Kawamoto, S., Kikuchi, M., et al. (2017). Exploring suicide risk factors among Japanese individuals: the largest case-control psychological autopsy study in Japan. *Asian Journal of Psychiatry*, *27*, 123–126.
	Hirokawa, S., Kawakami, N., Matsumoto, T., Inagaki, A., Eguchi, N., Tsuchiya, M., et al. (2012). Mental disorders and suicide in Japan: a nation-wide psychological autopsy case-control study. *Journal of Affective Disorders, 140*(2), 168–175.
	Kodaka, M., Matsumoto, T., Katsumata, Y., Akazawa, M., Tachimori, H., Kawakami, N., et al. (2014). Suicide risk among individuals with sleep disturbances in Japan: a case-control psychological autopsy study. *Sleep Medicine, 15*(4), 430–435.
e19	Kolves, K., Varnik, A., Tooding, L. M., & Wasserman, D. (2006). The role of alcohol in suicide: a case-control psychological autopsy study. *Psychological Medicine, 36*(7), 923–930.
	Kolves, K., Sisask, M., Anion, L., Samm, A., & Varnik, A. (2006). Factors predicting suicide among Russians in Estonia in comparison with Estonians: case-control study. *Croatian Medical Journal, 47*(6), 869–877.
	*Kolves, K., Varnik, A., Schneider, B., Fritze, J., & Allik, J. (2006). Recent life events and suicide: a case-control study in Tallinn and Frankfurt. *Social Science & Medicine, 62*(11), 2887–2896.
	*Schneider, B., Kolves, K., Blettner, M., Wetterling, T., Schnabel, A., & Varnik, A. (2009). Substance use disorders as risk factors for suicide in an Eastern and a Central European city (Tallinn and Frankfurt/Main). *Psychiatry Research, 165*(3), 263–272.
e20	Kurihara, T., Kato, M., Reverger, R., & Tirta, I.G.R. (2009). Risk factors for suicide in Bali: a psychological autopsy study. *BMC Public Health*, *9*: 327.
e21	Manoranjitham, S.D., Rajkumar, A.P., Thangadurai, P., Prasad, J., Jayakaran, R., & Jacob, K.S. (2010). Risk factors for suicide in rural south India. *British Journal of Psychiatry, 196*(1), 26–30.
e22	Martiello, M.A., Boncompagni, G., Lacangellera, D., & Corlito, G. (2019). Risk factors for suicide in rural Italy: a case-control study. *Social Psychiatry and Psychiatric Epidemiology, 54*(5), 607–616.
e23	Morales, E.J.M., & Martinez, M.I.G. (2010). Risk factors for suicide in Nariño, Colombia: a matched case-control study. *Revista Colombiana De Psiquiatría, 39*(2), 291–312.
e24	Nicolas, C., Seguin, M., DiMambro, M., & Desrumaux, P. (2016). Work difficulties and suicide: comparative study of life trajectories. *Annales Medico-Psychologiques, 174*(7), 544–550.
	Seguin, M., Renaud, J., Lesage, A., Robert, M., & Turecki, G. (2011). Youth and young adult suicide: a study of life trajectory. *Journal of Psychiatric Research, 45*(7), 863–870.
e25	Overholser, J. C., Braden, A., & Dieter, L. (2012). Understanding suicide risk: identification of high-risk groups during high-risk times. *Journal of Clinical Psychology, 68*(3), 349–361.
e26	Owens, C., Booth, N., Briscoe, M., Lawrence, C., & Lloyd, K. (2003). Suicide outside the care of mental health services: a case-controlled psychological autopsy study. *Crisis*, *24*(3), 113–121.
e27	Page, A., Morrell, S., Hobbs, C., Carter, G., Dudley, M., Duflou, J., & Taylor, R. (2014). Suicide in young adults: psychiatric and socio-economic factors from a case-control study. *BMC Psychiatry*, *14*: 68.

e28	Palacio, C., Valencia, J., Diago, J., Zapata, C., Lopez, G., Ortiz, J., & Lopez, M. (2007). Identification of suicide risk factors in Medellin, Colombia: a case-control study of psychological autopsy in a developing country. *Archives of Suicide Research, 11*(3), 297–308.
	Garcia-Valencia, J., Palacio-Acosta, C., Diago, J., Zapata, C., Lopez, G., Ortiz, J., & Lopez, M. (2008). Adverse life events and suicide: a case-control study of psychological autopsy in Medellin, Colombia. *Revista Colombiana De Psiquiatriia*, *37*(1), 11–28.
e29	Politakis, V.A., Pregelj, P., Videtic Paska, A., & Zupanc, T. (2017). Association between alcohol abuse, childhood adverse events and suicide. *Zdravniski Vestnik, 86*(10), 365–372.
e30	Rasouli, N., Malakouti, S.K., Rezaeian, M., Saberi, S.M., Nojomi, M., De Leo, D., & Ramezani-Farani, A. (2019). Risk factors of suicide death based on psychological autopsy method; a case-control study. *Archives of Academic Emergency Medicine, 7*(1): e50.
e31	Ross, V., Kolves, K., & De Leo, D. (2017). Beyond psychopathology: a case-control psychological autopsy study of young adult males. *International Journal of Social Psychiatry, 63*(2), 151–160.
e32	Schneider, B., Wetterling, T., Sargk, D., Schneider, F., Schnabel, A., Maurer, K., & Fritze, J. (2006). Axis I disorders and personality disorders as risk factors for suicide. *European Archives of Psychiatry and Clinical Neuroscience, 256*(1), 17–27.
	Muller, B., Georgi, K., Schnabel, A., & Schneider, B. (2009). Does sport have a protective effect against suicide? *Epidemiology and Psychiatric Sciences, 18*(4), 331–335.
	Schneider, B., Bartusch, B., Schnabel, A., & Fritze, J. (2005). Age and gender: confounders for axis I disorders as risk factors for suicide. *Psychiatrische Praxis, 32*(4), 185–194.
	Schneider, B., Georgi, K., Weber, B., Schnabel, A., Ackermann, H., & Wetterling, T. (2006). Risk factors for suicide in substance-related disorders. *Psychiatrische Praxis, 33*(2), 81–87.
	Schneider, B., Grebner, K., Schnabel, A., Hampel, H., Georgi, K., & Seidler, A. (2011). Impact of employment status and work-related factors on risk of completed suicide: a case-control psychological autopsy study. *Psychiatry Research*, *190*(2-3), 265–270.
	Schneider, B., Schnabel, A., Weber, B., Frolich, L., Maurer, K., & Wetterling, T. (2005). Nicotine use in suicides: a case-control study. *European Psychiatry, 20*(2), 129–136.
	Schneider, B., Schnabel, A., Wetterling, T., Bartusch, B., Weber, B., & Georgi, K. (2008). How do personality disorders modify suicide risk? *Journal of Personality Disorders, 22*(3), 233–245.
	Schneider, B., Wetterling, T., Georgi, K., Bartusch, B., Schnabel, A., & Blettner, M. (2009). Smoking differently modifies suicide risk of affective disorders, substance use disorders, and social factors. *Journal of Affective Disorders, 112*(1-3), 165–173.
	*Kolves, K., Varnik, A., Schneider, B., Fritze, J., & Allik, J. (2006). Recent life events and suicide: a case-control study in Tallinn and Frankfurt. *Social Science & Medicine, 62*(11), 2887–2896.
	*Schneider, B., Kolves, K., Blettner, M., Wetterling, T., Schnabel, A., & Varnik, A. (2009). Substance use disorders as risk factors for suicide in an Eastern and a Central European city (Tallinn and Frankfurt/Main). *Psychiatry Research, 165*(3), 263–272.
e33	Tong, Y., & Phillips, M.R. (2010). Cohort-specific risk of suicide for different mental disorders in China. *British Journal of Psychiatry*, *196*(6), 467–472.
	Phillips, M.R., Yang, G., Zhang, Y., Wang, L., Ji, H., & Zhou, M. (2002). Risk factors for suicide in China: a national case-control psychological autopsy study. *Lancet, 360*(9347), 1728–1736.
	Li, X.Y., Phillips, M.R., Zhang, Y.P., Xu, D., & Yang, G.H. (2008). Risk factors for suicide in China's youth: a case-control study. *Psychological Medicine, 38*(3), 397–406.
e34	Vijayakumar, L., & Rajkumar, S. (1999). Are risk factors for suicide universal? A case-control study in India. *Acta Psychiatrica Scandinavica*, *99*(6), 407–411.

e35	Zhang, J., Conwell, Y., Zhou, L., & Jiang, C. (2004). Culture, risk factors and suicide in rural China: a psychological autopsy case control study. *Acta Psychiatrica Scandinavica*, *110*(6), 430–437.
	Zhang, J., & Zhou, L. (2009). A case control study of suicides in China with and without mental disorder. *Crisis, 30*(2), 68–72.
e36	Zhang, J., Xiao, S., & Zhou, L. (2010). Mental disorders and suicide among young rural Chinese: a case-control psychological autopsy study. *American Journal of Psychiatry, 167*(7), 773–781.
	Jia, C.X., & Zhang, J. (2012). Global functioning and suicide among Chinese rural population aged 15–34 years: a psychological autopsy case-control study. *Journal of Forensic Sciences, 57*(2), 391–397.
	Kong, Y., & Zhang, J. (2010). Access to farming pesticides and risk for suicide in Chinese rural young people. *Psychiatry Research, 179*(2), 217–221.
	Li, Z., Zhang, J., Li, Z., & Zhang, J. (2012). Coping skills, mental disorders, and suicide among rural youths in China. *Journal of Nervous and Mental Disease, 200*(10), 885–890.
	Lin, L., & Zhang, J. (2017). Impulsivity, mental disorder, and suicide in rural China. *Archives of Suicide Research, 21*(1), 73–82.
	Lin, L., Zhang, J., Zhou, L., & Jiang, C. (2016). The relationship between impulsivity and suicide among rural youths aged 15–35 years: a case-control psychological autopsy study. *Psychology, Health & Medicine, 21*(3), 330–337.
	Lyu, J., & Zhang, J. (2021). Suicide means, timing, intent and behavior characteristics of the suicides with schizophrenia. *Psychiatry Research, 306*: 114267.
	Lyu, J., Ding, L., & Zhang, J. (2021). Relationship and effects of community problems on anxiety and suicide: a case-control psychological autopsy study. *Journal of Nervous and Mental Disease, 209*(1), 17–22.
	Sun, L., & Zhang, J. (2015). Coping skill as a moderator between negative life events and suicide among young people in rural China. *Journal of Clinical Psychology, 71*(3), 258–266.
	Sun, L., Zhang, J., & Liu, X. (2015). Insomnia symptom, mental disorder and suicide: a case-control study in Chinese rural youths. *Sleep and Biological Rhythms, 13*(2), 181–188.
	Sun, L., Zhang, J., Lamis, D.A., & Wang, Y. (2021). Risk assessment on suicide death and attempt among Chinese rural youths aged 15-34 years. *International Journal of Environmental Research and Public Health, 18*(24): 13362.
	Zhang, J. (2014). The gender ratio of Chinese suicide rates: an explanation in Confucianism. *Sex Roles, 70*(3), 146–154.
	Zhang, J., & Jia, C. (2011). Suicidal intent among young suicides in rural China. *Archives of Suicide Research, 15*(2), 127–139.
	Zhang, J., & Li, Z. (2013). The association between depression and suicide when hopelessness is controlled for. *Comprehensive Psychiatry, 54*(7), 790–796.
	Zhang, J., & Lin, L. (2014). The moderating effects of impulsivity on Chinese rural young suicide. *Journal of Clinical Psychology, 70*(6), 579–588.
	Zhang, J., & Lin, L. (2015). The moderating effect of social support on the relationship between impulsivity and suicide in rural China. *Community Mental Health Journal, 51*(5), 585–590.
	Zhang, J., & Ma, Z. (2012). Patterns of life events preceding the suicide in rural young Chinese: a case control study. *Journal of Affective Disorders, 140*(2), 161–167.
	Zhang, J., Kong, Y., Gao, Q., & Li, Z. (2013). When aspiration fails: a study of its effect on mental disorder and suicide risk. *Journal of Affective Disorders, 151*(1), 243–247.

	Zhang, J., Lamis, D.A., & Yuanyuan, K. (2012). Measuring Chinese psychological traits and social support with Western developed instruments in psychological autopsy studies. *Journal of Clinical Psychology, 68*(12), 1313–1321.
	Zhang, J., Li, N., Tu, X.M., Xiao, S., & Jia, C. (2011). Risk factors for rural young suicide in China: a case-control study. *Journal of Affective Disorders, 129*(1-3), 244–251.
	Zhang, J., Liu, X., & Fang, L. (2019). Combined effects of depression and anxiety on suicide: a case-control psychological autopsy study in rural China. *Psychiatry Research, 271*, 370–373.
	Zhang, J., Wieczorek, W., Conwell, Y., Tu, X.M., Wu, B.Y., Xiao, S., et al. (2010). Characteristics of young rural Chinese suicides: a psychological autopsy study. *Psychological Medicine, 40*(4), 581–589.
	Zhang, J., Wieczorek, W.F., Conwell, Y., & Tu, X.M. (2011). Psychological strains and youth suicide in rural China. *Social Science & Medicine, 72*(12), 2003–2010.
	Zhao, S., & Zhang, J. (2014). Suicide risks among adolescents and young adults in rural China. *International Journal of Environmental Research and Public Health, 12*(1), 131–145.
	Zhou, Q., Zhang, J., & Hennessy, D.A. (2019). The role of family absolute and relative income in suicide among Chinese rural young adults: mediation effects of social support and coping strain. *Journal of Public Health, 41*(3), 609–617.
e37	Zonda, T. (2006). One-hundred cases of suicide in Budapest: a case-controlled psychological autopsy study. *Crisis*, *27*(3), 125–129.

Note. The publication selected as the main reference for the respective study (table 1) is highlighted in grey.
* These two publications reported combined data for a portion of the German sample (n = 163 suicides and 163 controls) and a portion of the Estonian sample (limited to Tallinn; n = 156 suicides and 156 controls).

Figure S1. Study selection.

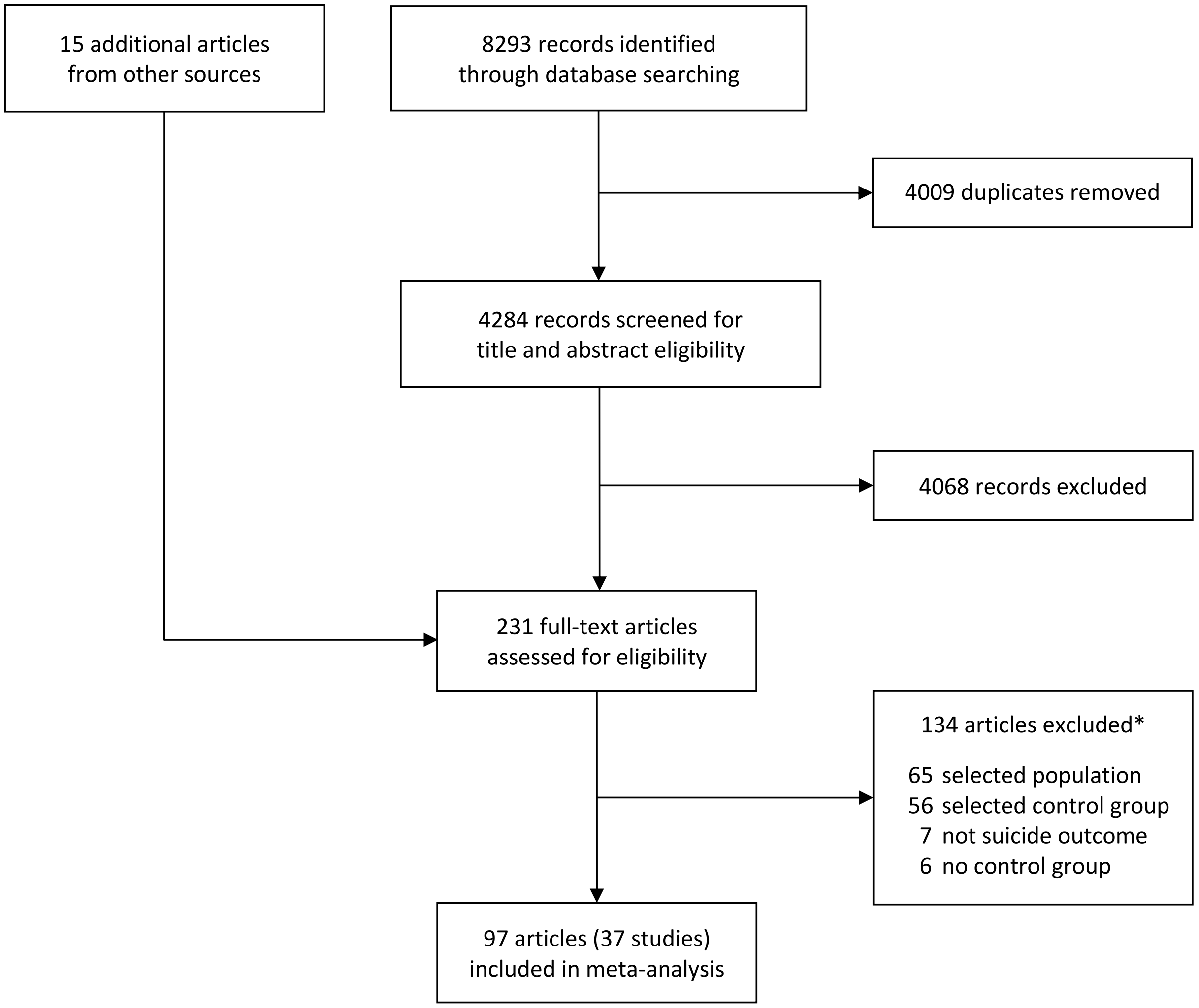

* Articles can be excluded for multiple reasons; numbers listed are based on the major reason for exclusion.

Favril L, *et al. Evid Based Ment Health* 2022; 25:148–155. doi: 10.1136/ebmental-2022-300549

Table S4. Risk of bias scores.

Study	Case definition	Represent-ativeness	Selection of controls	Definition of controls	Comparability	Ascertainment of exposure	Method of ascertainment	Non-response	Total (out of 9)
Almasi et al. (2009)	1	0	1	0	2	0	0	1	5
Altindag et al. (2005)	1	1	1	1	2	1	1	1	9
Anton-San-Martin et al. (2013)	1	1	1	1	2	0	1	0	7
Appleby et al. (1999)	0	0	1	1	2	1	1	0	6
Arafat et al. (2021)	1	1	1	1	2	0	0	1	7
Beautrais (2001)	1	1	1	1	2	0	0	1	7
Chachamovich et al. (2015)	1	0	1	1	0	0	1	0	4
Chen et al. (2006)	1	1	1	1	2	0	1	0	7
Cheng et al. (2000)	1	1	1	1	2	1	1	0	8
de la Vega Sanchez et al. (2020)	1	1	0	1	0	0	1	0	4
De Leo et al. (2013)	1	1	0	1	0	1	1	1	6
Foster et al. (1999)	1	1	1	1	2	0	1	0	7
Gururaj et al. (2004)	1	1	1	1	2	0	1	0	7
Jia et al. (2014)	1	1	1	1	2	0	1	0	7
Jollant et al. (2014)	1	0	0	1	2	0	1	0	5
Khan et al. (2008)	1	1	1	1	2	0	1	0	7
Kim et al. (2003)	1	1	1	1	2	0	1	1	8
Kodaka et al. (2017)	1	1	1	1	2	0	1	0	7
Kolves et al. (2006)	1	0	1	1	2	1	0	1	7
Kurihara et al. (2009)	1	1	1	1	2	1	1	1	9
Manoranjitham et al. (2010)	1	0	1	1	2	0	1	0	6
Martiello et al. 2019	1	1	1	1	2	0	1	0	7
Morales & Martinez (2010)	1	1	1	1	2	0	1	0	7
Nicolas et al. (2016)	1	1	1	1	0	0	1	0	5
Overholser et al. (2012)	1	1	1	0	0	0	1	0	4
Owens et al. (2003)	1	0	1	1	2	0	1	0	6
Page et al. (2014)	1	1	1	1	2	0	0	1	7
Palacio et al. (2007)	1	1	0	1	2	0	1	0	6
Politakis et al. (2017)	1	1	0	0	2	0	0	0	4
Rasouli et al. (2019)	1	1	1	1	2	0	1	0	7
Ross et al. (2017)	1	1	1	1	0	1	1	0	6
Schneider et al. (2006)	1	1	1	1	2	0	0	0	6
Tong & Phillips (2010)	1	1	1	1	0	0	1	0	5
Vijayakumar & Rajkumar (1999)	1	1	1	1	2	0	1	0	7
Zhang et al. (2004)	1	0	1	0	2	0	0	1	5
Zhang et al. (2010)	1	1	1	1	2	0	1	0	7
Zonda (2006)	1	0	1	1	2	0	1	1	7

Note. Risk of bias scores based on the Newcastle-Ottawa (NOS) scale.

Table S5. Prevalence for the strongest risk factors within each domain.

Risk factor	Prevalence (%)	
	Case group	Control group
Any mental disorder	71.1	22.2
Past-month adverse life events	58.8	18.4
Family history of mental disorder	48.4	22.4
Social isolation	43.9	13.7

Favril L, *et al. Evid Based Ment Health* 2022; 25:148–155. doi: 10.1136/ebmental-2022-300549

Table S6. Comparison between crude (OR) and adjusted (aOR) odds ratios.

Risk factor	*k*	OR (95% CI)	aOR (95% CI)	% Δ
Previous suicide attempt	8	18.4 (12.8–26.5)	10.2 (6.4–16.0)	-45
History of self-harm	10	18.1 (13.2–24.6)	9.1 (6.0–13.7)	-50
Any mental disorder	13	12.1 (7.7–19.0)	7.7 (5.1–11.6)	-36
Depression	8	11.3 (5.1–25.1)	11.0 (4.9–24.6)	-3
Any personality disorder	4	10.7 (2.5–45.7)	4.2 (1.2–14.2)	-61
Schizophrenia spectrum disorder	5	7.4 (3.2–17.2)	5.6 (2.3–13.3)	-24
Smoking	3	5.2 (3.2–8.6)	3.4 (2.0–5.8)	-35
Family history suicidal behaviour	4	5.1 (3.8–6.9)	4.3 (2.4–7.5)	-16
Unemployment	9	4.9 (3.0–7.9)	3.5 (2.0–6.0)	-29
Social isolation	4	4.8 (3.1–7.5)	4.0 (2.5–6.4)	-17
Not religious	3	4.0 (1.1–13.8)	5.9 (2.9–12.0)	+48
Alcohol use disorder	5	3.8 (2.3–6.3)	2.6 (1.8–3.7)	-32
Anxiety disorder	6	3.5 (2.0–6.0)	3.1 (1.1–8.8)	-11
Single/not married	7	3.4 (2.2–5.2)	3.0 (2.1–4.4)	-12
Work/school-related conflict	3	3.2 (2.4–4.4)	3.9 (1.6–10.0)	+22
Low education	7	3.0 (2.3–3.8)	2.1 (1.6–2.8)	-30
Physical illness	7	2.6 (2.0–3.4)	2.3 (1.4–3.9)	-12
Low income	3	2.6 (1.6–4.3)	2.5 (1.6–4.0)	-4
Low SES	3	2.6 (1.6–4.1)	2.4 (1.6–4.6)	-8
Substance use disorder	6	2.3 (1.5–3.6)	2.4 (1.8–3.1)	+4
Living alone	6	1.9 (0.9–3.8)	1.1 (0.7–1.9)	-42

Note. Risk factors are ranked in order of crude effect sizes. SES = socioeconomic status.

Table S7. Meta-regression and publication bias analyses for risk factors based on 10 or more samples.

	High-income		Deceased control		Direct interview		Sample size		Proportion men		Risk of bias		PB
	B	SE	B	SE	B	SE	B	SE	B	SE	B	SE	*p*
Social isolation	0.46	0.50	—	—	0.48	0.55	-0.0017	0.0009	**0.045**	**0.017**	-0.20	0.24	**0.001**
Unemployment	0.34	0.29	-0.43	0.34	0.51	0.37	0.0001	0.0004	-0.002	0.010	-0.04	0.13	**0.015**
Low education	0.21	0.26	0.13	0.39	0.27	0.35	0.0002	0.0003	0.010	0.009	**-0.19**	**0.09**	0.865
Single/not married	0.39	0.35	**-0.68**	**0.30**	0.30	0.43	0.0001	0.0009	**-0.016**	**0.009**	0.16	0.12	0.138
Living alone	0.52	0.44	**-1.14**	**0.33**	-0.25	0.54	**-0.0010**	**0.0004**	-0.009	0.014	**0.42**	**0.16**	**0.044**
Not religious	0.24	0.55	0.29	0.61	0.41	0.92	-0.0004	0.0029	0.009	0.022	0.25	0.20	0.836
Family history of suicide	-0.33	0.48	-0.35	0.59	0.69	0.70	0.0008	0.0012	0.011	0.027	0.04	0.21	0.119
Any mental disorder	0.06	0.29	**-0.73**	**0.35**	0.01	0.45	-0.0001	0.0005	-0.001	0.007	0.17	0.13	**0.009**
Depression	**-1.00**	**0.39**	-0.17	0.46	-0.98	0.62	**0.0011**	**0.0006**	0.008	0.010	-0.06	0.20	**0.071**
Schizophrenia	0.35	0.62	**-1.41**	**0.46**	**1.58**	**0.69**	0.0003	0.0007	**0.023**	**0.012**	**0.48**	**0.26**	0.104
Substance use disorder	0.24	0.39	**-0.84**	**0.30**	0.47	0.39	**-0.0006**	**0.0003**	0.002	0.007	0.10	0.18	**0.006**
Alcohol use disorder	-0.01	0.41	**-1.14**	**0.35**	0.53	0.36	0.0004	0.0006	-0.003	0.009	0.14	0.20	0.677
Anxiety disorder	-0.18	0.50	0.20	0.46	-0.66	0.41	0.0000	0.0007	-0.003	0.008	0.26	0.22	0.357
Any personality disorder	-0.76	0.64	**-0.87**	**0.29**	-0.09	0.42	-0.0031	0.0026	-0.004	0.006	**0.27**	**0.13**	**0.006**
Psychiatric treatment	0.59	0.42	-0.47	0.40	—	—	-0.0005	0.0004	-0.007	0.020	-0.01	0.24	0.256
History of self-harm	0.07	0.46	0.02	0.47	**1.43**	**0.73**	**0.0014**	**0.0007**	0.002	0.018	0.08	0.17	0.081
Previous suicide attempt	-0.22	0.48	0.31	0.48	1.52	0.97	**0.0017**	**0.0007**	-0.005	0.019	0.13	0.17	0.205
Physical illness	-0.09	0.21	-0.42	0.29	-0.03	0.26	-0.0001	0.0003	-0.018	0.012	0.06	0.11	0.555
Relationship conflict	-0.83	0.45	-0.66	0.50	-0.07	0.54	0.0001	0.0007	-0.024	0.031	0.22	0.15	0.835
Financial problems	0.16	0.45	-0.20	0.54	-0.60	0.42	0.0002	0.0017	-0.009	0.028	0.24	0.18	**0.061**
Work/school conflict	-0.91	0.59	0.03	0.74	-0.71	0.49	0.0005	0.0009	-0.025	0.035	0.37	0.24	0.565

Note. Significant effects ($p < 0.10$) are marked in bold. PB = publication bias (Egger's test).

Table S8. Subgroup analyses by type of control group.

Risk factor	Living control group			Deceased control group		
	k	OR (95% CI)	I^2	*k*	OR (95% CI)	I^2
Sociodemographic						
Social isolation	10	4.0 (2.1–7.7)	95	0	—	—
Unemployment	19	4.2 (2.9–6.3)	80	6	2.7 (1.5–4.8)	75
Low SES	4	2.8 (1.8–4.2)	0	0	—	—
Low education	17	2.7 (2.0–3.5)	66	2	—	—
Single/not married*	16	2.9 (2.1–3.9)	74	5	1.4 (1.0–1.8)	0
Low income	4	2.4 (1.4–4.2)	80	2	—	—
Living alone*	11	3.1 (2.1–4.6)	59	4	1.0 (0.6–1.5)	47
Not religious	9	2.1 (1.1–3.8)	82	3	2.7 (1.9–4.0)	1
Not having children	4	1.4 (1.1–1.8)	0	2	—	—
Family history						
Mental disorder	6	5.2 (1.9–14.1)	82	0	—	—
Suicide	11	4.0 (2.3–6.9)	75	3	2.8 (1.1–7.2)	68
Suicide attempt	5	2.9 (1.3–6.6)	83	2	—	—
Clinical						
Any mental disorder*	22	15.8 (11.3–22.2)	72	6	7.4 (5.1–10.7)	62
Depression	15	11.1 (7.2–17.2)	59	7	10.0 (4.4–22.9)	90
Schizophrenia*	11	14.0 (7.0–28.0)	51	6	3.5 (2.2–5.5)	13
Bipolar disorder	5	4.9 (1.3–18.1)	61	3	4.6 (2.1–10.1)	0
Substance use disorder*	15	4.9 (3.8–6.4)	29	6	1.9 (1.4–2.6)	41
Alcohol use disorder*	15	3.9 (3.2–4.8)	5	3	1.3 (2.3–3.2)	85
Drug use disorder	4	5.0 (1.7–14.4)	37	2	—	—
Anxiety disorder	12	2.3 (1.5–3.7)	54	5	2.8 (1.5–5.4)	55
Dysthymia	6	2.4 (1.4–4.1)	36	0	—	—
Any personality disorder	10	7.4 (5.5–9.8)	0	3	4.7 (1.6–13.6)	60
Borderline PD	6	9.2 (5.6–15.0)	0	1	—	—
Paranoid PD	4	6.2 (3.4–11.4)	0	1	—	—
Dependent PD	4	6.1 (2.5–15.1)	0	0	—	—
Avoidant PD	4	4.2 (1.2–24.1)	77	1	—	—
Antisocial PD	5	3.4 (2.0–6.1)	0	0	—	—
Psychiatric treatment*	7	13.3 (8.0–22.3)	58	4	7.4 (4.6–11.9)	0
History of self-harm	19	10.3 (5.7–18.3)	77	7	10.2 (5.2–19.9)	80
Previous suicide attempt	15	7.6 (4.1–14.3)	74	7	10.2 (5.2–19.9)	80

Favril L, *et al. Evid Based Ment Health* 2022; 25:148–155. doi: 10.1136/ebmental-2022-300549

Smoking	4	4.3 (2.3–7.9)	67	0	—	—
Physical illness	14	3.1 (2.5–3.8)	18	2	—	—
Adverse life events						
Relationship conflict*	8	5.9 (3.6–9.4)	75	2	—	—
Legal problems	5	5.4 (2.3–13.1)	77	1	—	—
Family-related conflict	8	4.7 (1.9–11.4)	93	1	—	—
Abuse/victimisation	6	3.5 (2.4–5.0)	0	0	—	—
Financial problems	12	3.0 (2.0–4.4)	65	3	2.5 (1.0–6.6)	70
Early separation	4	2.7 (1.6–4.5)	5	0	—	—
Work/school conflict	9	1.8 (1.1–2.9)	87	2	—	—
Bereavement	4	0.7 (0.2–3.1)	92	3	2.4 (1.1–5.3)	32
Within past month	6	11.1 (6.4–19.1)	33	1	—	—
Within past 6 months*	2	—	—	1	—	—
Within past 3 months	4	2.9 (1.6–5.0)	72	0	—	—

Note. SES = socioeconomic status; PD = personality disorder.
* Heterogeneity between subgroups significant at the 0.10 level.

Favril L, *et al. Evid Based Ment Health* 2022; 25:148–155. doi: 10.1136/ebmental-2022-300549

Table S9. Sensitivity analysis excluding studies in which controls acted as their own informants.

Risk factor	Excluded (sensitivity analysis)			Included (main analysis)		
	k	OR (95% CI)	I^2	*k*	OR (95% CI)	I^2
Sociodemographic						
Social isolation	8	3.6 (1.8–6.9)	91	10	4.0 (2.1–7.7)	95
Unemployment	21	3.3 (2.3–4.5)	77	25	3.8 (2.7–5.2)	79
Low education	16	2.6 (2.0–3.4)	67	19	2.7 (2.1–3.5)	64
Single/not married	18	2.3 (1.8–3.0)	68	21	2.4 (1.8–3.2)	74
Living alone	12	2.5 (1.5–4.0)	80	15	2.3 (1.5–3.4)	77
Not religious	11	2.1 (1.3–3.6)	78	12	2.2 (1.4–3.5)	77
Not having children	5	1.2 (0.8–1.6)	57	6	1.3 (0.9–1.8)	61
Family history						
Family history of suicide	12	3.4 (2.1–5.5)	75	14	3.7 (2.3–5.7)	73
Clinical						
Any mental disorder	24	13.2 (9.6–18.2)	77	28	13.1 (9.9–17.4)	76
Depression	19	12.5 (8.1–19.5)	78	22	11.0 (7.3–16.5)	77
Schizophrenia spectrum	14	5.9 (3.5–9.8)	51	17	7.8 (4.5–13.5)	63
Bipolar disorder	5	3.7 (1.4–9.5)	27	8	4.6 (2.1–10.1)	33
Substance use disorder	16	3.2 (2.2–4.6)	76	21	3.7 (2.8–5.0)	71
Alcohol use disorder	14	2.8 (1.9–4.1)	70	18	3.2 (2.3–4.4)	71
Anxiety disorder	13	3.0 (2.0–4.7)	44	17	2.5 (1.7–3.5)	52
Dysthymia	3	4.0 (2.3–7.1)	0	6	2.4 (1.4–4.1)	36
Any personality disorder	10	7.8 (4.5–13.8)	55	13	6.8 (4.7–9.8)	42
Borderline PD	5	9.7 (4.9–19.1)	0	7	9.0 (5.6–14.4)	0
Paranoid PD	3	4.5 (1.8–11.7)	0	5	6.2 (3.5–11.2)	0
Dependent PD	2	—	—	4	6.1 (2.5–15.1)	0
Avoidant PD	3	3.2 (0.5–21.4)	74	5	3.9 (1.4–11.1)	69
Antisocial PD	3	3.6 (1.9–6.8)	0	5	3.4 (2.0–6.1)	0
History of self-harm	23	8.9 (5.7–13.9)	77	26	10.1 (6.6–15.6)	77
Previous suicide attempt	20	7.7 (4.8–12.4)	78	22	8.5 (5.3–13.4)	77
Smoking	1	—	—	4	4.3 (2.3–7.9)	67
Physical illness	13	3.0 (2.4–3.7)	12	16	2.9 (2.4–3.6)	19
Adverse life events						
Relationship conflict	8	5.0 (3.4–7.4)	54	10	5.0 (3.3–7.6)	73
Legal problems	5	4.8 (2.1–11.3)	80	6	4.8 (2.4–9.4)	75
Family-related conflict	6	4.7 (1.5–14.6)	93	9	4.5 (2.0–10.3)	92

Abuse/victimisation	5	3.4 (2.2–5.1)	7	6	3.5 (2.4–5.0)	0
Financial problems	11	3.6 (2.1–6.0)	70	15	2.8 (2.0–4.0)	65
Work/school conflict	7	2.4 (1.4–4.1)	78	11	1.8 (1.1–2.8)	84
Bereavement	5	2.2 (1.0–5.2)	79	7	1.2 (0.5–3.1)	86
Within past month	6	11.8 (8.3–16.8)	0	7	10.4 (7.1–15.3)	19
Within past 6 months	2	—	—	3	5.3 (1.8–15.9)	91
Within past 3 months	1	—	—	4	2.9 (1.6–5.0)	72

Note. Risk factors not listed in this table were based solely on studies that used proxy informants for controls. PD = personality disorder.

Figure S2. Funnel plot for social isolation.

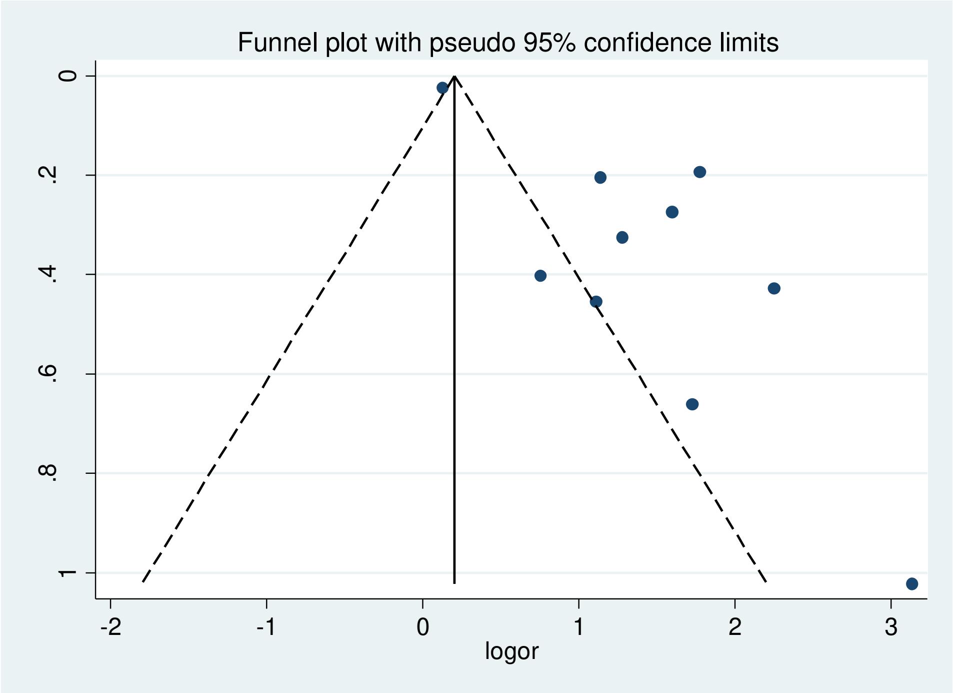

Figure S3. Funnel plot for unemployment.

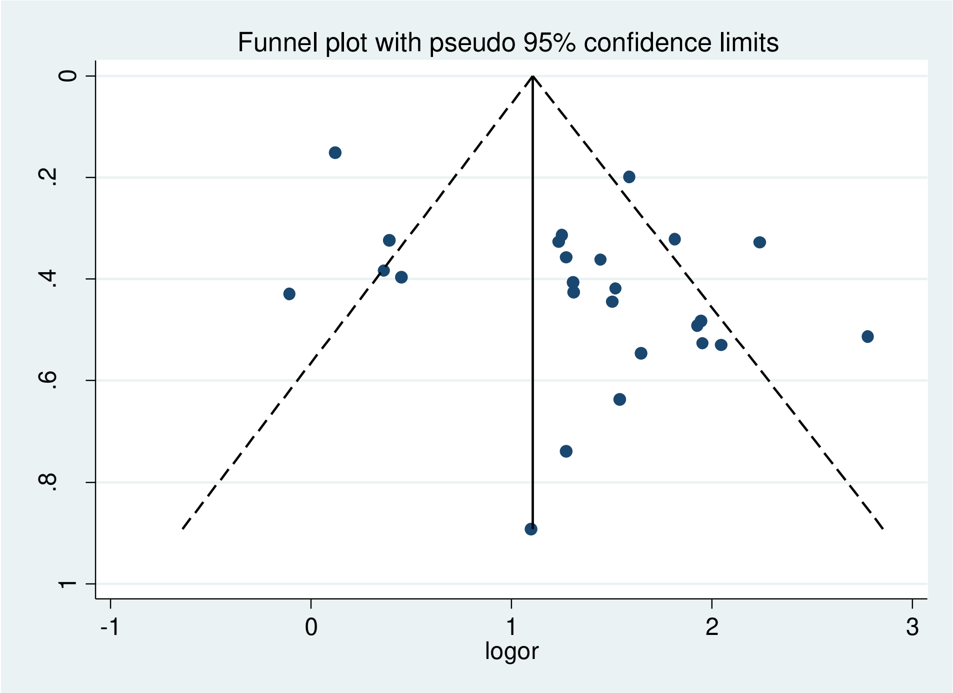

Favril L, *et al. Evid Based Ment Health* 2022; 25:148–155. doi: 10.1136/ebmental-2022-300549

Figure S4. Funnel plot for living alone.

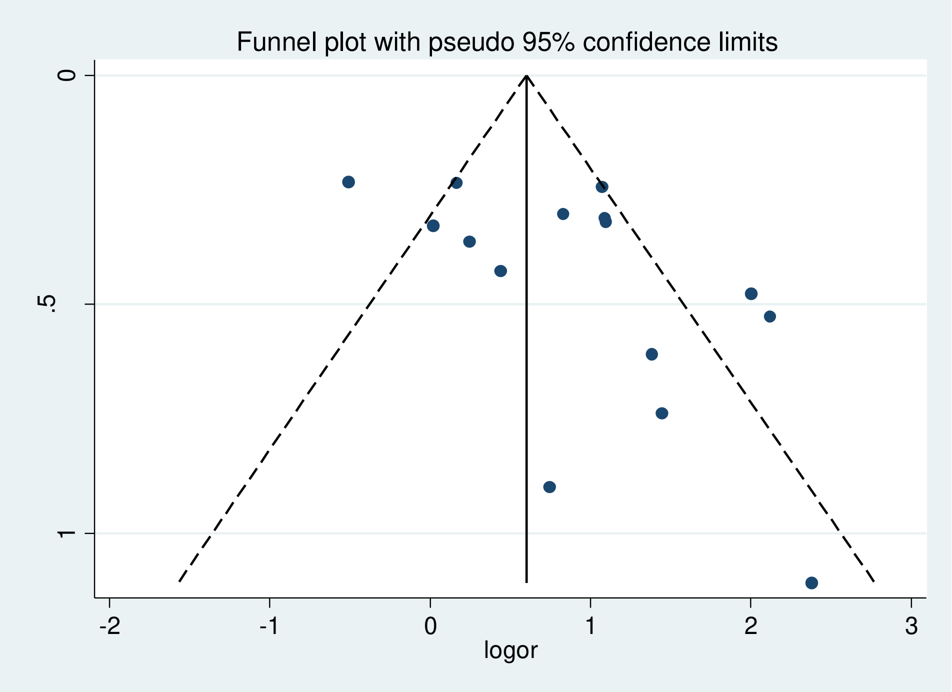

Favril L, *et al. Evid Based Ment Health* 2022; 25:148–155. doi: 10.1136/ebmental-2022-300549

Figure S5. Funnel plot for any mental disorder.

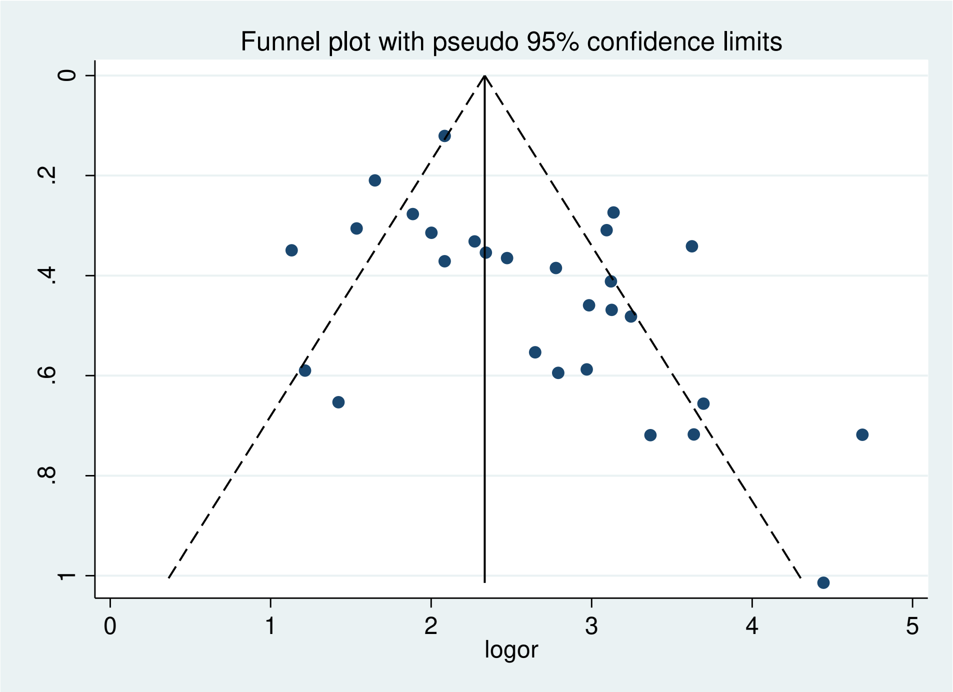

Favril L, *et al. Evid Based Ment Health* 2022; 25:148–155. doi: 10.1136/ebmental-2022-300549

Figure S6. Funnel plot for depression.

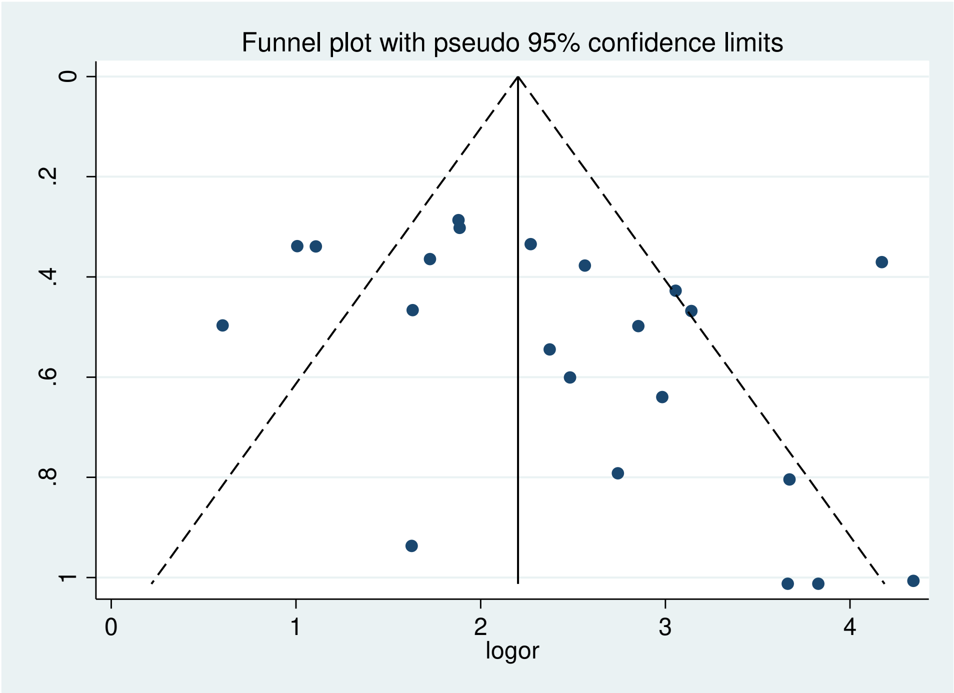

Favril L, *et al. Evid Based Ment Health* 2022; 25:148–155. doi: 10.1136/ebmental-2022-300549

Figure S7. Funnel plot for substance use disorder.

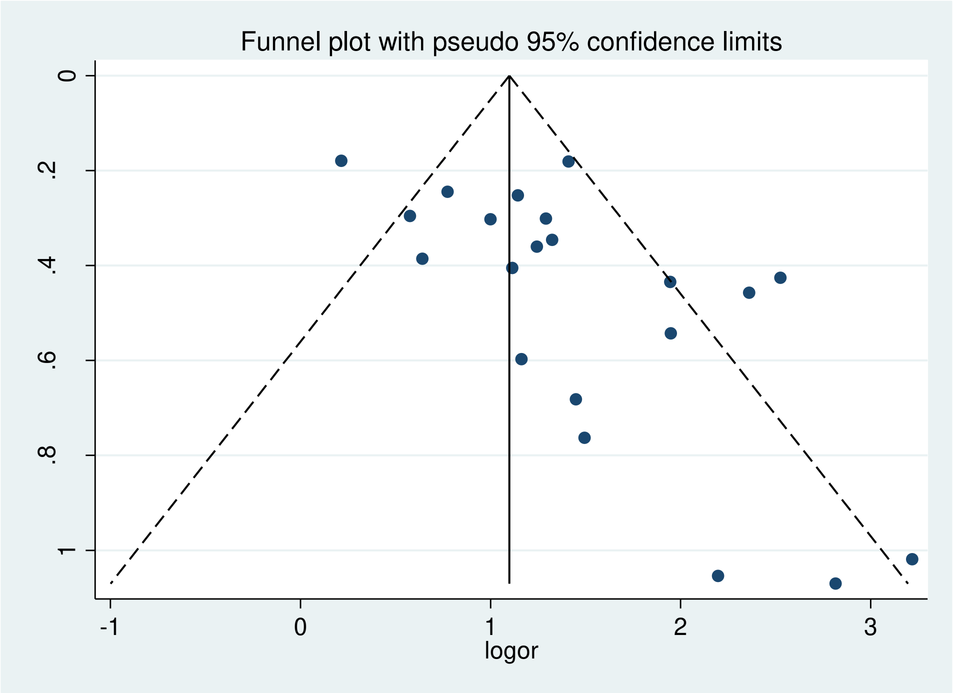

Figure S8. Funnel plot for any personality disorder.

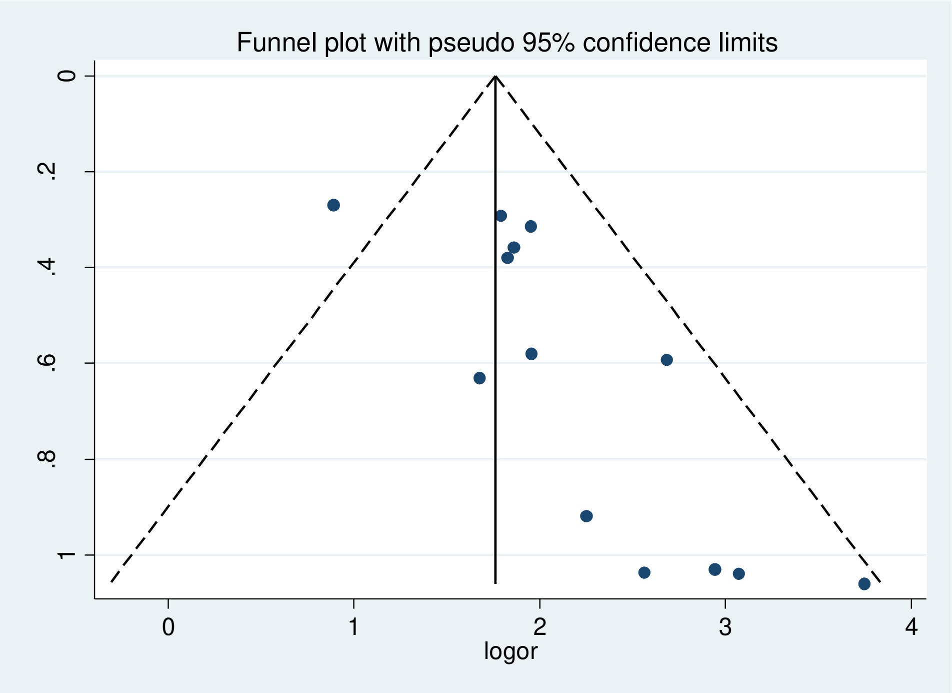

Figure S9. Funnel plot for financial problems.

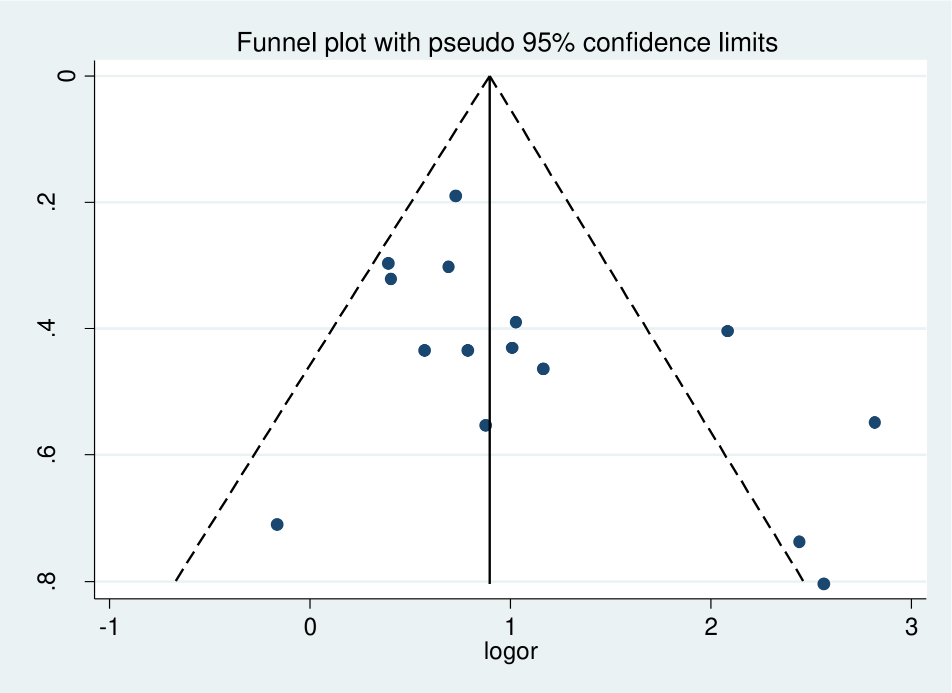

Supplement: Supplementary data [file ebmental-2022-300549supp001.pdf]
